# Country-level pandemic risk and preparedness classification based on COVID-19 data: A machine learning approach

**DOI:** 10.1371/journal.pone.0241332

**Published:** 2020-10-28

**Authors:** Jordan J. Bird, Chloe M. Barnes, Cristiano Premebida, Anikó Ekárt, Diego R. Faria

**Affiliations:** 1 Aston Robotics, Vision, and Intelligent Systems Lab (ARVIS), School of Engineering and Applied Science, Aston University, Birmingham, United Kingdom; 2 Department of Electrical and Computer Engineering, Institute of Systems and Robotics, University of Coimbra, Coimbra, Portugal; Helmholtz Centre for Infection Research (HZI), GERMANY

## Abstract

In this work we present a three-stage Machine Learning strategy to country-level risk classification based on countries that are reporting COVID-19 information. A K% binning discretisation (K = 25) is used to create four risk groups of countries based on the risk of transmission (coronavirus cases per million population), risk of mortality (coronavirus deaths per million population), and risk of inability to test (coronavirus tests per million population). The four risk groups produced by K% binning are labelled as ‘low’, ‘medium-low’, ‘medium-high’, and ‘high’. Coronavirus-related data are then removed and the attributes for prediction of the three types of risk are given as the geopolitical and demographic data describing each country. Thus, the calculation of class label is based on coronavirus data but the input attributes are country-level information regardless of coronavirus data. The three four-class classification problems are then explored and benchmarked through leave-one-country-out cross validation to find the strongest model, producing a Stack of Gradient Boosting and Decision Tree algorithms for risk of transmission, a Stack of Support Vector Machine and Extra Trees for risk of mortality, and a Gradient Boosting algorithm for the risk of inability to test. It is noted that high risk for inability to test is often coupled with low risks for transmission and mortality, therefore the risk of inability to test should be interpreted first, before consideration is given to the predicted transmission and mortality risks. Finally, the approach is applied to more recent risk levels to data from September 2020 and weaker results are noted due to the growth of international collaboration detracting useful knowledge from country-level attributes which suggests that similar machine learning approaches are more useful prior to situations later unfolding.

## 1 Introduction

According to the Future of Humanity Institute there is a 2.05% chance that mankind will go extinct by the year 2100, through either a natural or engineered pandemic [1]. If there is one lesson to learn from the ongoing COVID-19 Coronavirus (SARS-CoV-2) pandemic, it is that we were not prepared. The virus initially spread rapidly across the globe, mortality began to rise, and countries desperately struggled to test their citizens for the virus once it became known that many infectious carriers of it show no noticeable symptoms [2–4]. This suggests three main risk factors to be observant of: the initial risk of transmission due to varying factors such as, for example, population density [5] and international travel [6]; the risk of mortality due to ageing populations [7] and underlying health issues [8, 9]; and finally the risk of a country not being able to test citizens aptly and thus producing possibly under-reported measures of the previous two [10].

Machine learning has shown success in contributing to research during the COVID-19 pandemic. Health service data trend models have shown to aid in classification of the virus [11, 12], vaccine design [13], estimation of cases, deaths, and recoveries [14, 15], simulating what could have happened if *‘lockdown’* was not instituted [16], and also simulating behaviour of the spread of the disease by prior knowledge from other locations [17].

In this work, we devise a machine learning based strategy to predict three-fold risk at the country-level: (i) risk of transmission, (ii) risk of mortality, and (iii) risk of inability to test. Through these three quantifiable measures, preparedness and risk can be assessed, providing some quantitative reasoning behind global decisions, should another deadly disease grip our species again. Our main contribution is the exploration of the idea that country-level demographic and geopolitical attributes can aid in the classification of pandemic risk and preparedness in terms of transmission, mortality, and an inability to test (which the previous two depend on, since testing allows for accurate measurements of transmission and mortality). In order to do this, various supervised learning classifiers are explored in order to discern how much useful information these country-level attributes carry for the classification of these three risks.

We note that the classification problems are difficult, where many powerful techniques achieve unsatisfactory scores on the dataset, scoring around 10-20% higher than an approximate 25% random guess on the dataset, showing that learning useful rules from the data is not an easy task. This is not unexpected, since the classes have not been directly derived from the data used to predict them, rather, they have been derived from COVID-19 statistics and then given as classes for country-level demographic and geopolitical information.

Due to this, strategies of linear searching and genetic optimisation are also followed in order to achieve more accurate results. Although results are varied, the fact that all final models achieve much higher than 25% accuracy We formulate the problem as a 4-class problem. (which would be achieved via a random guess), shows that the geopolitical and demographic attributes at the country-level do carry predictive ability when it comes to pandemic risk and preparedness. The final models chosen are characterised by high classification accuracy for the risks of transmission, mortality, and inability to test, and are trained with no prior knowledge of the new coronavirus pandemic (other than the class). This may allow for generalisation to classify a nation’s risk in the early days of a future pandemic.

The remainder of this work is organised as follows: Section 2 details the method followed with Subsection 2.1 describing machine learning approaches in particular. Section 3 presents the results for the risk of transmission (3.1), the risk of mortality (3.2) and the risk of inability to test (3.3). Finally, the limitations of the study are described, future work is suggested, and the study is concluded in Section 4.

## 2 Method

Firstly, a numerical risk score is calculated for all countries that record publicly the number of COVID-19 cases, deaths, and tests performed, which are grouped into four classes after being collected from [18] Formalised on the 12th May 2020, updated experiments for newer data can be found in Section 3.7, with the relative ordering based on the three metrics with regards to population (cases, deaths, and tests per million). The risk classes are low, medium-low, medium-high and high for each type of risk. As defined in other works [19–21], discretisation of the continuous features into bins is performed by the *K*% method in which *K* = 25 (equal frequency binning), resulting in four close-to-equal classes, with the difference being that the highest risk class is a minor 1.2% larger than the other three classes. Future work aims to explore other methods of discretisation, whereas this work initially focuses on the machine learning pipeline on the basis of equal class error weighting.

COVID-19 data are then removed, and the attributes to complement the country-level classes are the following: UN Region [22], 2020 Population Estimate [18], Median Age [18], Population Density per *km*^2^ [18], Urban Population % [18], Urban Population total [18], Nursing and midwifery personnel per 10,000 (most recently recorded) [23], Medical doctors per 10,000 (most recently recorded) [23], Tobacco prevalence 2016 [24], Obesity prevalence 2016 [25], Gross Domestic Product 2019 [22], Land area *KM*^2^ [22], Net Migration [22], Infant mortality per 1,000 births [22], Literacy rate % [22], Arable land % [22], Crop land % [22], Other land % [22], Climate classification type [22], Birth rate per 1,000 [22], Death rate per 1,000 [22], GDP expenditure on Agriculture [22], GDP expenditure on Industry [22] and GDP expenditure on Services [22]. Since some countries are not recorded by The World Health Organisation, figures for Nursing, midwifery and medical doctors personnel per 10,000 people from Hong Kong are collected from an alternative source [26]. Missing data which occurred mostly for tobacco prevalence, was given as ‘-1’, which flags as an attribute that the data have not been collected (which could in itself provide useful information).

**Fig 1:**
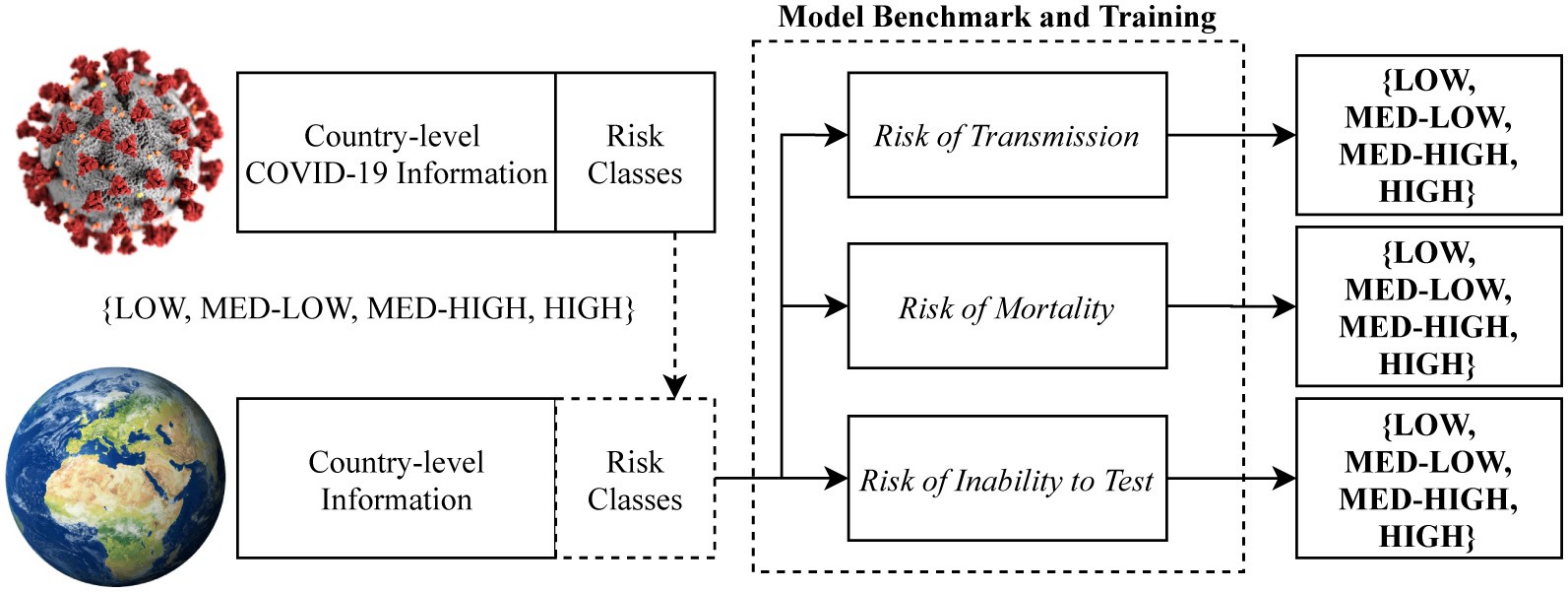
Overall diagram of the experiment. Risk is calculated with COVID-19 information but is then classified via country-level geopolitical and demographic attributes.

The classification problem of risk is therefore formulated based on prior knowledge of the pandemic in terms of class only, but the attributes to attempt to classify them are purely country-level information regardless of number of cases, deaths and other coronavirus specific data. Thus the problem becomes a pandemic risk and preparedness classification problem based on demographic and geopolitical attributes only. We aim for a generalisable model, which can be applied to the future state of countries, should another potential pandemic begin prior to any meaningful measurements being available. The method is illustrated in Fig 1.

Following this, a set of machine learning models are tasked with predicting a country’s risk class by learning from all other countries in a process of Leave One Out cross-validation [27], which is performed for all three types:

1. *Risk of Transmission*—derived from COVID-19 cases per million population
2. *Risk of Mortality*—derived from COVID-19 deaths per million population
3. *Risk of Inability to Test*—derived from COVID-19 tests performed per million population

Finally, the best models for each risk factor are organised into a predictive framework, which produces an output for the three risks.

**Fig 2:**
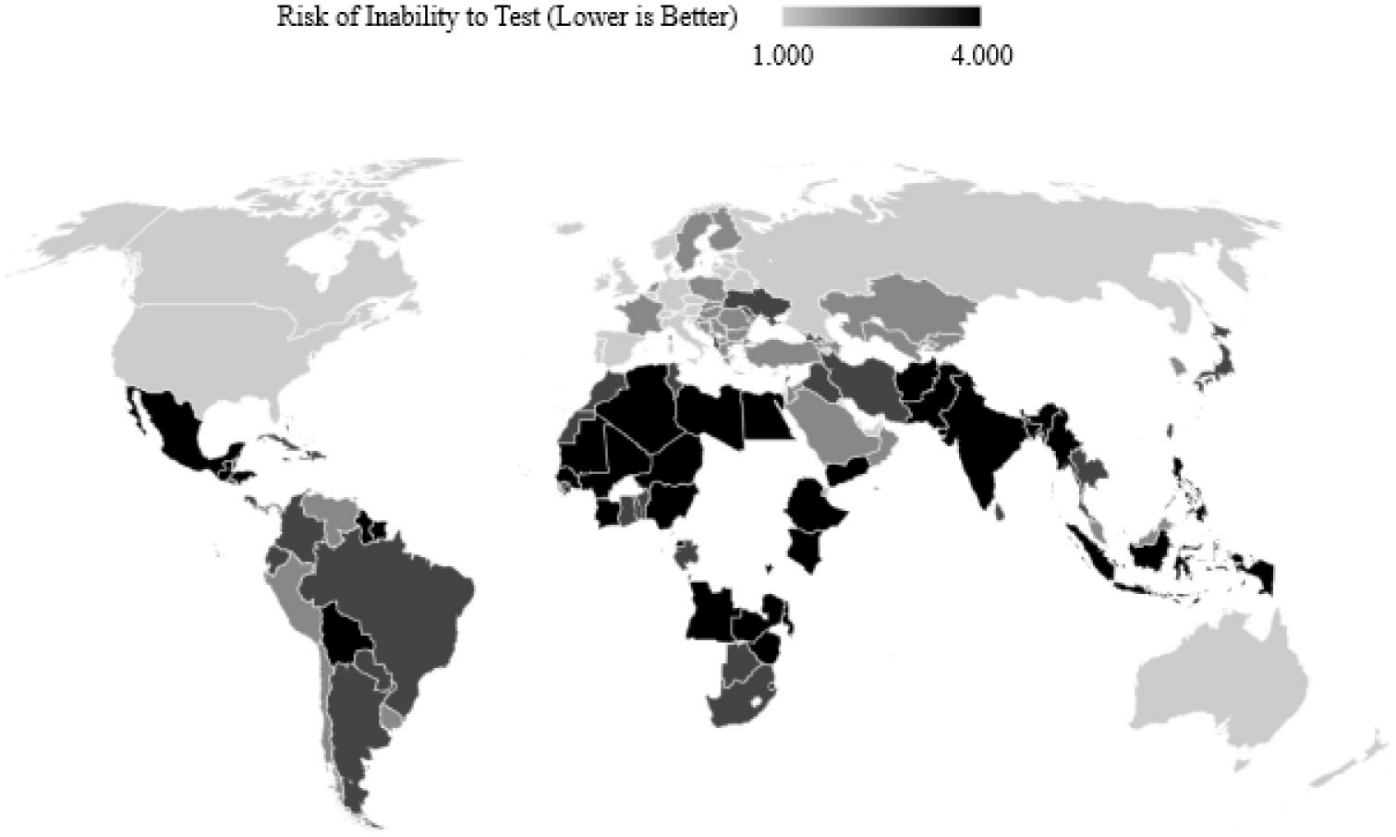
A map of COVID-19 tests performed per million people divided into four classes with outlier countries removed. Note that this map is juxtapose to those shading transmission and mortality.

**Fig 3:**
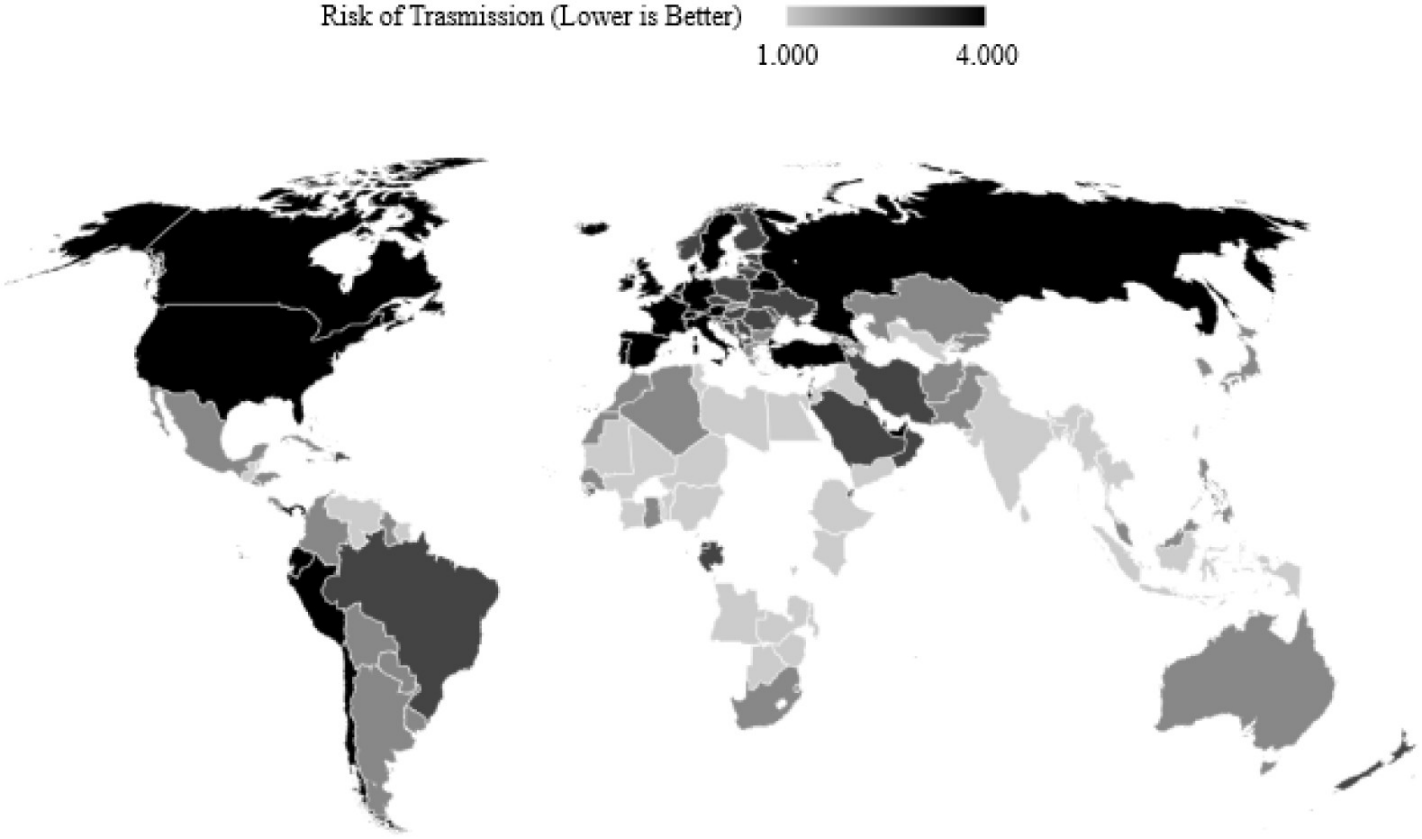
A map of COVID-19 cases per million people divided into four classes with outlier countries removed. Note that many countries at “low risk” by number of cases are at “high risk” for inability to test.

**Fig 4:**
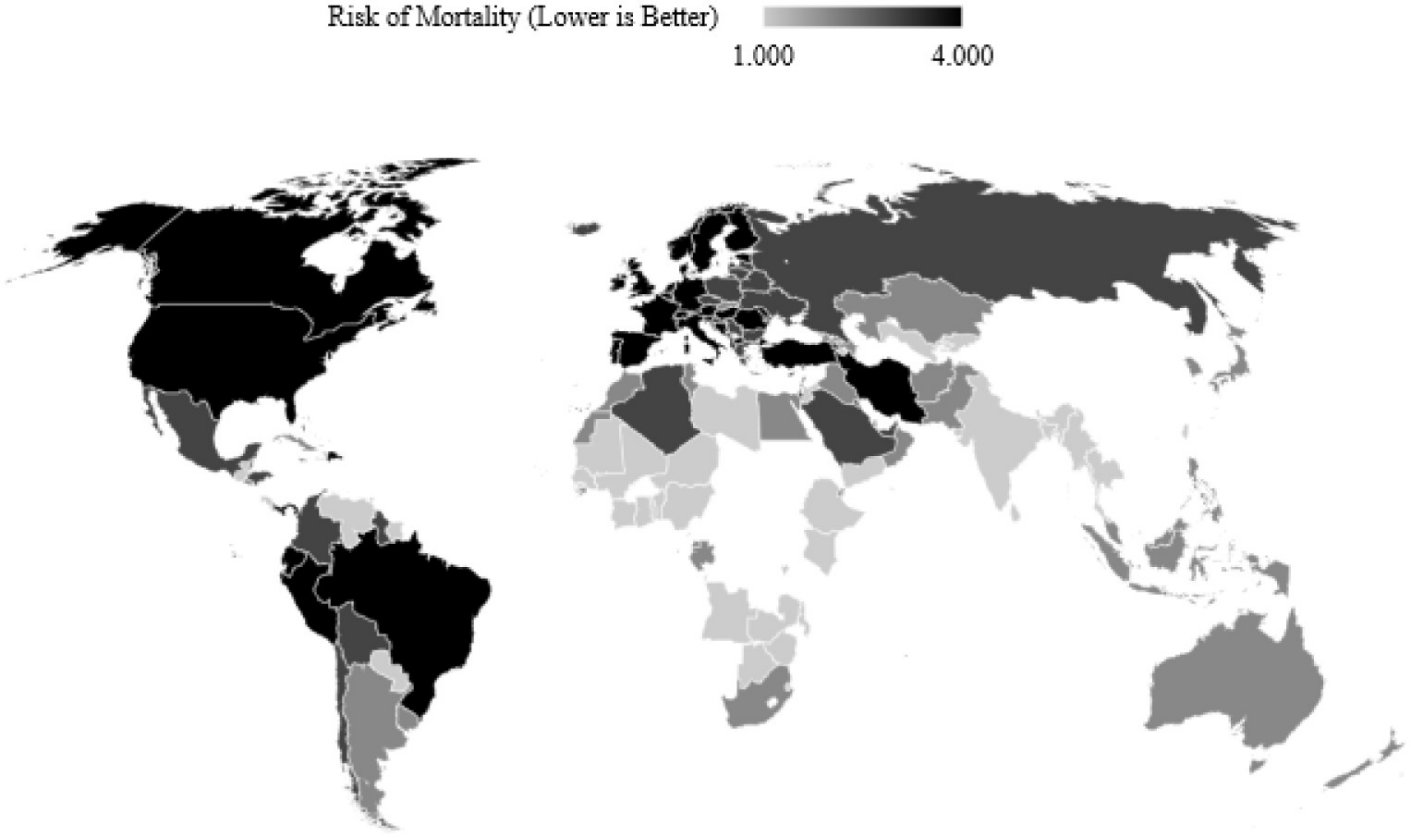
A map of COVID-19 deaths per million people divided into four classes with outlier countries removed. Note that many countries at “low risk” by number of deaths are at “high risk” for inability to test.

**Fig 5:**
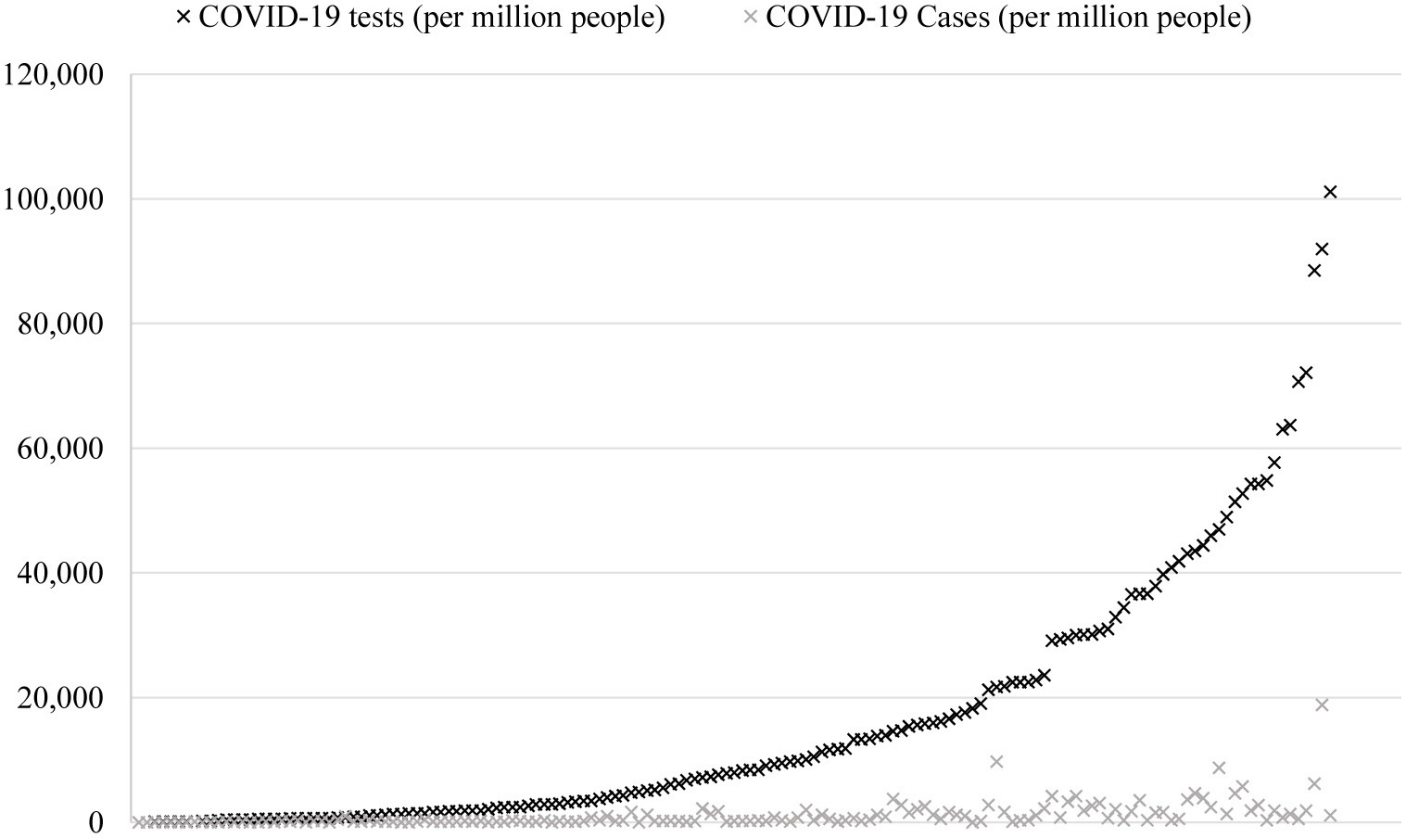
Comparison of the distributions of tests performed per million people and confirmed cases per million people. Number of fatalities per million people show a similar behaviour w.r.t tests but are omitted for readability purposes. Position on the X axis denotes each country.

Since testing is taken into account, countries that have not reported testing data cannot be considered, but are later classified by the model generalised on those countries that do. A three-fold machine learning approach is proposed following observing the maps for the three separate risk quarters in Figs 2, 3 and 4 which show the discretised inability to test risk, transmission risk, and mortality risk respectively. We note that the countries with seemingly fewer cases have performed far fewer tests as can be observed in Fig 5, and thus this should be considered an important observation; to give examples, the nation of Yemen has performed only 4 tests per million population, Burundi 24 per million, Malawi 70 per million, and Angola 91 per million—the fewest four of any country on earth. In comparison, Spain has performed 52,781 tests per million population, Italy 43,112, the United Kingdom 29,566 and the United States of America 29,147 COVID-19 tests per million population. Additionally, Fig 5 shows that the growth of cases and testing tend to increase alongside one another. That is, a country with more cases will test more, and as such will have more confirmed cases, since the larger number of tests have identified more cases. The data for the two experiments were accessed on 12^*th*^ May 2020 and 16^*th*^ September 2020.

### 2.1 Machine learning approaches

Trained with the strategy of Leave One Out cross-validation (LOO CV) where every country’s risk is predicted based on learning from all other countries, a set of supervised classification models are benchmarked. This section details the models focused upon, and the methods used to search for others. The metrics reported following the models described in this section are mean classification accuracy due to close-to-equal class balance [28] (Low, Med-low, Med-High are equal and High is minimally larger by a factor of 1.2%) and high variance often observed due to the nature of LOO CV [29, 30].

Decision Trees are tree structures, where each internal node represents a condition based on attributes that allows splitting the data and leaf nodes represent class labels [31]. A Random Decision Forest (RDF) [32], used in this study, creates multiple random decision trees, where each decision tree votes on the class of the input data object, and the predicted class is that, which receives the majority vote. Splitting of the trees is based on information gain:

$$\begin{array}{c} IG(T,X)=E(T)-E(T,X), \end{array}$$ (1)

where IG is the observed difference in information entropy, which is expressed in Eq (2), that is, the nodes split data based on reducing the randomness of object class distribution.

K-Nearest Neighbours (KNN) is similar to an RDF in that the prediction is derived by a majority vote. The voters, rather than decision trees, are the data objects within the observations that are closest in terms of *n*-dimensional Euclidean space where *n* is the number of attributes.

Gradient Boosting [33] forms an ensemble of weak learners (decision trees) and aims to minimise a loss function via a forward stage-wise additive method. In these classification problems, deviance is minimised. At each stage, four trees (*n* = *classes*) are fit on the negative gradient of the multinomial deviance loss function, or cross-entropy loss [34, 35]:

$$\begin{array}{c} -\sum_{c=1}^{K}i_{x,y}\text{log}\left(p_{x,y}\right), \end{array}$$ (2)

where, for *K* classes, *i* is a binary indicator of whether the prediction that class *y* is the class of observed data *x* is correct, and finally *p* is the probability that aforementioned data *x* belongs to the class label *y*. XGBoost [36] differs slightly in that it penalises trees, leaves are shrunk proportionally, and extra randomisation is implemented.

Naïve Bayes is a probabilistic classifier that aims to find the posterior probability for a number of different hypotheses and selecting the most likely case. Bayes’ Theorem is given as:

$$\begin{array}{c} P\left(h|d\right)=\frac{P(d|h)P(h)}{P(d)}, \end{array}$$ (3)

where *P*(*h*|*d*) is the posterior probability of hypothesis *h* given the data *d*, *P*(*d*|*h*) is the conditional probability of data *d* given that the hypothesis *h* is true. *P*(*h*) *i.e*., the prior, is the probability of hypothesis *h* being true and *P*(*d*) = *P*(*d*|*h*)*P*(*h*) is the probability of the data. Naïvety in the algorithm is due to the assumption that each probability value is conditionally independent for a given target, calculated as $P\left(d|h\right)=\prod_{i=1}^{n}P\left(d_{i}|h\right)$ where *n* is the number of attributes/features.

Linear Discriminant Anaylsis (LDA), based on Fisher’s linear discriminant [37], is a statistical method that aims to find a linear combination of input features that separate classes of data objects, and then use those separations as feature selection (opting for the linear combination) or classification (placing prediction objects within a separation). Classes *k* ∈ {1, …, *K*} are assigned priors ${\hat{\pi }}_{k}$ ($\sum_{i=1}^{k}{\hat{\pi }}_{k}=1$). With Eq (3) in mind, maximum-a-posteriori probability is thus calculated as:

$$\begin{array}{c} G\left(x\right)=\text{arg}\;\underset{k}{\text{max}}\;\text{Pr}\left(\text{G}=\text{k}|\text{X}=\text{x}\right)=\text{arg}\;\underset{\text{k}}{\text{max}}\;{\text{f}}_{\text{k}}\left(\text{x}\right)\pi_{\text{k}}, \end{array}$$ (4)

where *f*_*k*_(*x*) is the density of *X* conditioned on *k*:

$$\begin{array}{c} f_{k}\left(x\right)={|2\pi \Sigma_{k}|}^{-1/2}\text{exp}(-\frac{1}{2}{\left(x-\mu_{k}\right)}^{T}\Sigma_{k}^{-1}\left(x-\mu_{k}\right)), \end{array}$$ (5)

Σ_*k*_ is the covariance matrix for samples of class *k* and class covariance matrices are assumed to be equal. The class discriminant function *δ*_*k*_(*x*) is given as:

$$\begin{array}{c} \delta_{k}\left(x\right)=x^{T}\Sigma^{-1}\mu_{k}-\frac{1}{2}\mu_{k}^{T}\Sigma^{-1}\mu_{k}+\text{log}\;\pi_{k}, \end{array}$$ (6)

where ${\hat{\mu }}_{k}$ is the class mean, and finally classification is performed via

$$\begin{array}{c} G\left(x\right)=\text{arg}\;\underset{k}{\text{max}}\;\delta_{k}\left(x\right). \end{array}$$ (7)

Quadratic Discriminant Analysis (QDA) is an algorithm that uses a quadratic plane to separate classes of data objects. Following the example of LDA, QDA estimates the covariance matrices of each class rather than operating on the assumption that they are the same. QDA follows LDA with the exception that:

$$\begin{array}{c} \delta_{k}\left(x\right)=-\frac{1}{2}\text{log}\left|\Sigma_{k}\right|-\frac{1}{2}{\left(x-\mu_{k}\right)}^{T}\Sigma_{k}^{-1}\left(x-\mu_{k}\right)+\text{log}\;\pi_{k}\;. \end{array}$$ (8)

Support Vector Machines (SVM) optimise a high dimensional hyperplane to best separate a set of data point by class by maximising the margin and minimising the empirical risk, and then predict new data points based on the distance vector measured from the hyperplane [38]. The optimisation of the hyperplane is to achieve the goal of maximising the average margins between the points and separator. Generation of a multi-class SVM is performed through Sequential Minimal Optimisation (SMO) [39] by breaking down the optimisation into smaller linearly-solvable sub-problems. For multipliers *a*, reduced constraints are given as:

$$\begin{array}{c} \begin{array}{c} 0\leq a_{1},a_{2}\leq C,\\ y_{1},a1+y_{2},a_{2}=k, \end{array} \end{array}$$ (9)

where there are data classes *y* and *k* are the negative of the sum over the remaining terms of the equality constraint.

Stacked Generalisation (Stacking) [40] is the process of training a machine learning algorithm to interpret the predictions of an ensemble of algorithms trained upon the dataset in a process of meta-learning. Generally, a stack can represent any kind of ensemble, but the interpretation algorithm is often Logistic Regression. It has been noted in multiple domains that Stacking often outperforms the individual models in the ensemble [41–43].

### 2.2 Initial observations

It was observed during experimentation that the classification problems were difficult, leading to many models achieving relatively bad results, *i.e*., the results outperformed an approximate 25% chance random guess by around 10-20% classification accuracy, with many state-of-the-art models predicting the wrong value more than half of the time (< 50%). The solutions explored to solve this are the following: A linear search is performed for Random Decision Forests (RDF) and K-Nearest Neighbours (KNN) from 10, 20, …, 1000 decision trees and 1, 2, …, 50 neighbours, respectively. Random Forests are often found to be powerful ML algorithms, and so an in-depth search is performed in order to maximise their ability. This is also followed for KNN since it is of low complexity and can thus be quickly benchmarked.

A genetic search is also performed via the Tree-based Pipeline Optimization Tool (TPOT) algorithm detailed in [44] with consideration to the whole Scikit-learn toolkit [45] Where not detailed in the previous section, more information is available on the models in [46]. TPOT is an algorithm that treats each machine learning operator as a Genetic Programming (GP) primitive which include, modified features, feature combinations, feature selections and dimensionality reductions, learning algorithms as well as their predictions (for exploration of ensembles). GP Trees were chosen since they best represented a machine learning pipeline and are implemented with the DEAP framework [47], and best solutions are selected by the Multi-objective NSGA-II algorithm [48] by aiming to increase classification accuracy while reducing minimising the number of machine learning operators as previously described. 5% of offspring produced by the best models cross-over with another through a process of one-point crossover, and the remaining offspring randomly mutate at a 33% chance of point, insertion, or shrinkage. Thus, the algorithm introduces and tunes ML operators with promising effect and removes operators that cause the results to degrade. Finally, the best machine learning pipeline is presented from the search.

To conclude, the method described in this section follows the process of manual exploration, linear search, and genetic programming in order to explore the best classification models for these problems in terms of classification accuracy. As previously described, accuracy is chosen as the metric of comparison since the datasets are closely balanced, and the drawback of LOO is high variance (large standard deviation due to binary per-fold results) while enabling classification model validation of a small dataset.

### 2.3 Implementation

All of the experiments in this paper were performed using the Scikit-learn toolkit [45] implemented in Python. The algorithms were executed on an Intel Core i7 Processor (3.7GHz).

Due to the large computational complexity when searching a problem space with LOO, the algorithm was executed three times with a population size of 10 for 10 generations, if a model scored lower than the manually or linearly explored models then it was discarded, and otherwise presented if it achieved a higher score. This decision was based on the fact that results for the three problems attained were only 49.67%, 43.79%, and 56.21%, and more robust models were required in order to provide accurate predictions.

## 3 Results

**Fig 6:**
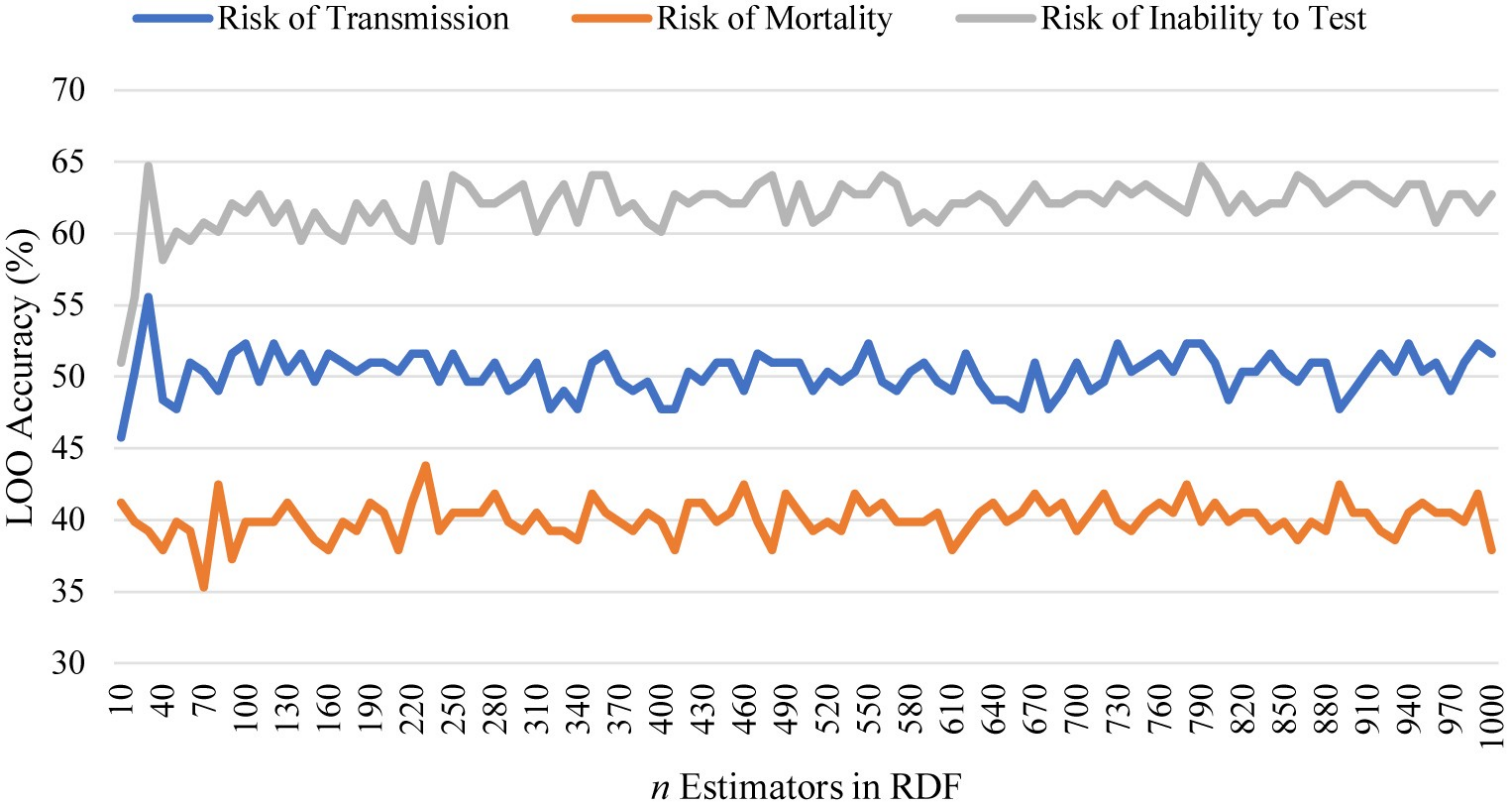
A linear search of RDF estimators for the three classification problems. Many of the solutions are weak due to the difficulty of the classification problem.

**Fig 7:**
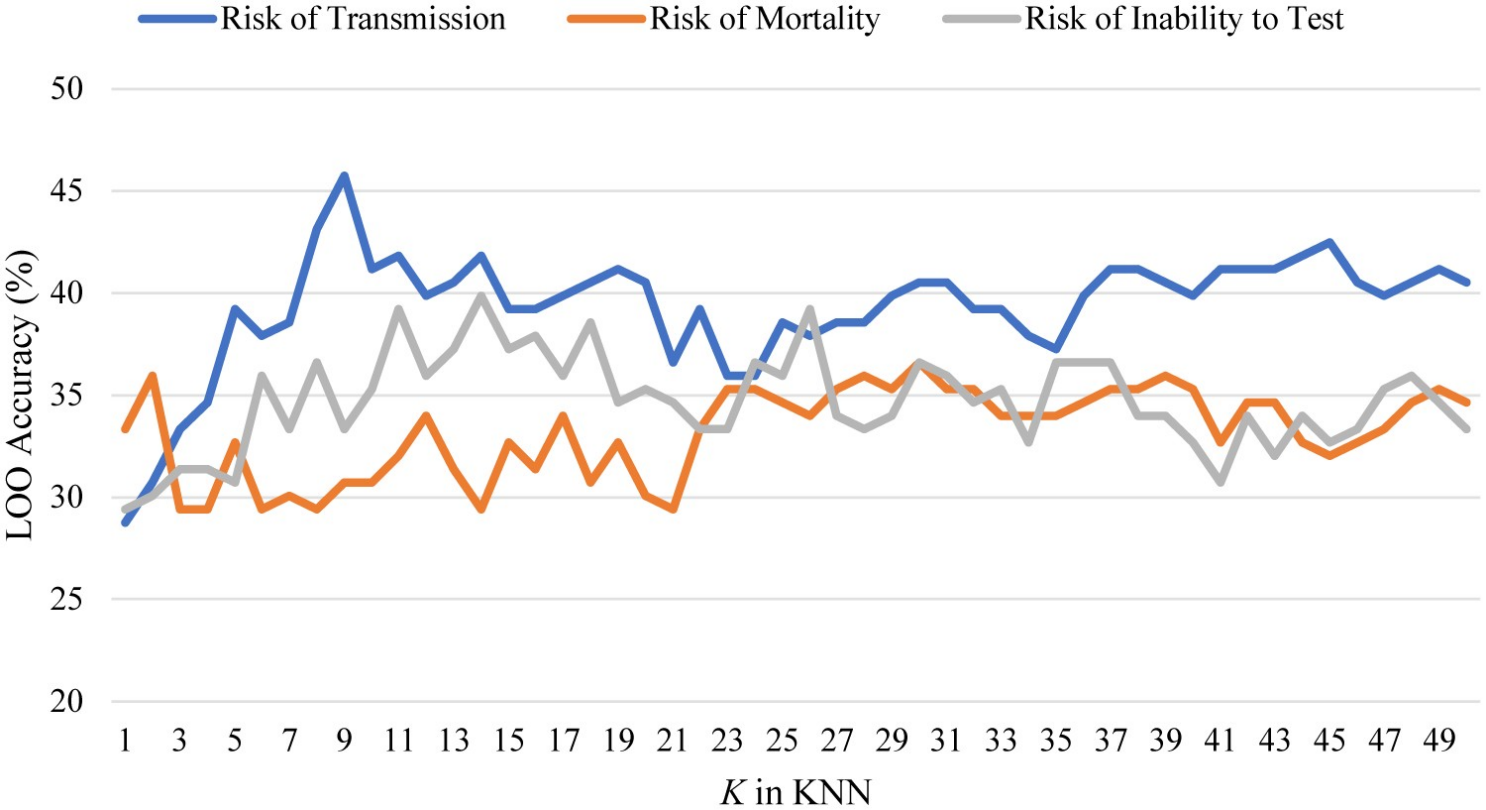
A linear search of KNN for the three classification problems. Results show that KNN is a weak solution for this problem.

In this section, the three sets of results are presented. For readability purposes, linear searches of RDF and KNN are presented as the same Figs (6) and (7).

### 3.1 Risk of transmission

Fig 6 shows the linear search for RDF estimators towards risk of transmission, where the best model was a forest 30 trees, which scored 55.6%. Fig 7 shows the linear search for KNN similarly, where the best model was *K* = 9 which scored 45.76%. In the RDF approach for the three different classification problems, risk of transmission always scored second, and first for KNN, albeit that the solutions presented were relatively weak.

**Fig 8:**
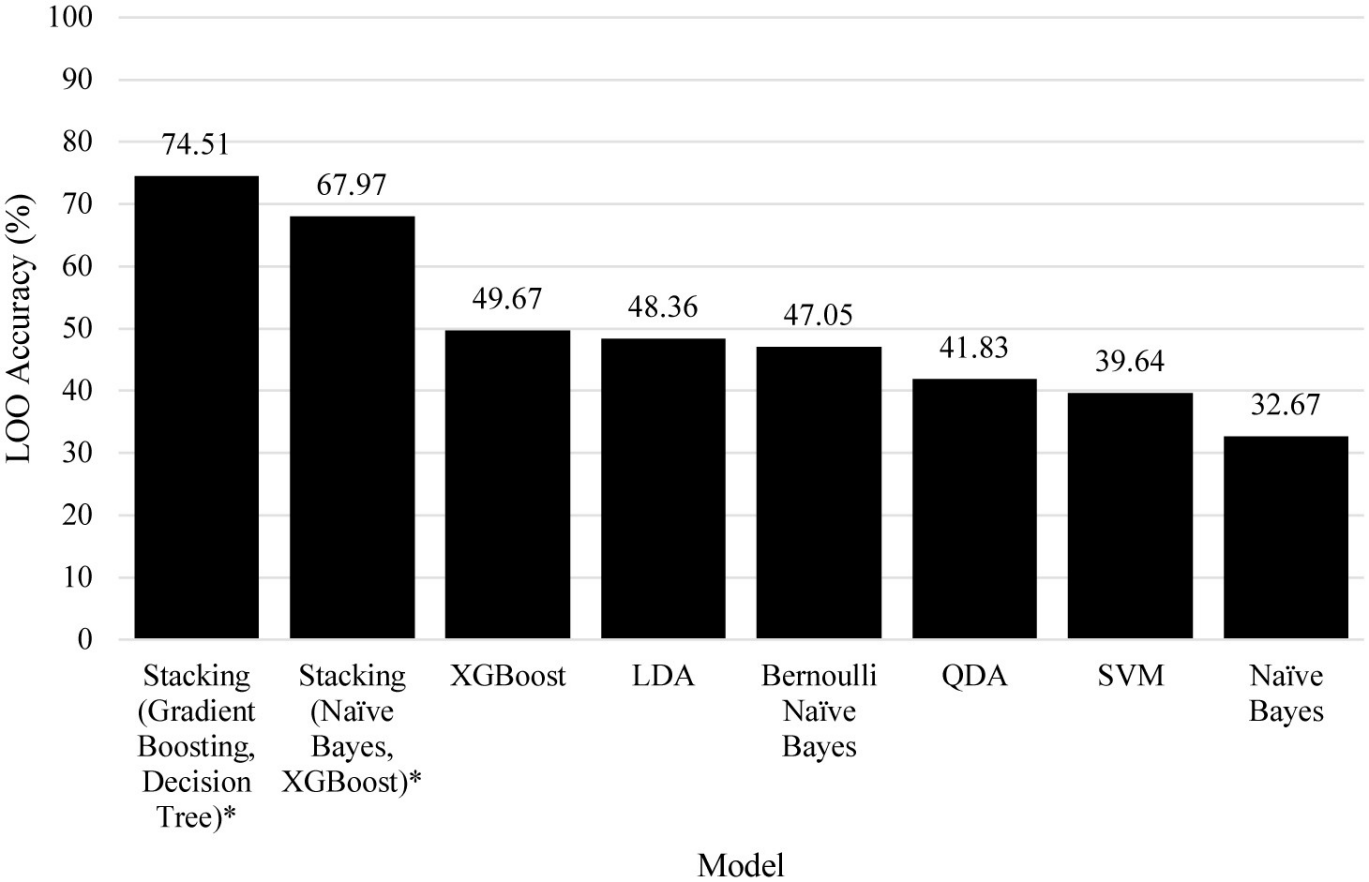
Comparison of models towards the risk of transmission, an asterisk denotes algorithms found by the genetic search algorithm.

Fig 8 details the other models explored for the classification of transmission risk. Likewise to the linear searches, many solutions were weak, achieving between only 32.67% and 49.67% for the four classes. The TPOT genetic search on the other hand suggested two relatively strong algorithms. Both were stacking algorithms, Stacking(Naïve Bayes, XGBoost) scored 67.97% accuracy and Stacking(Gradient Boosting, Decision Tree) scored the highest at 74.51% accuracy.

### 3.2 Risk of mortality

The linear searches for RDF and KNN are shown in Figs 6 and 7, respectively. The best RDF was a forest of 230 trees which scored 43.8%, and the best KNN had a value of *K* = 30 which scored 36.6%.

**Fig 9:**
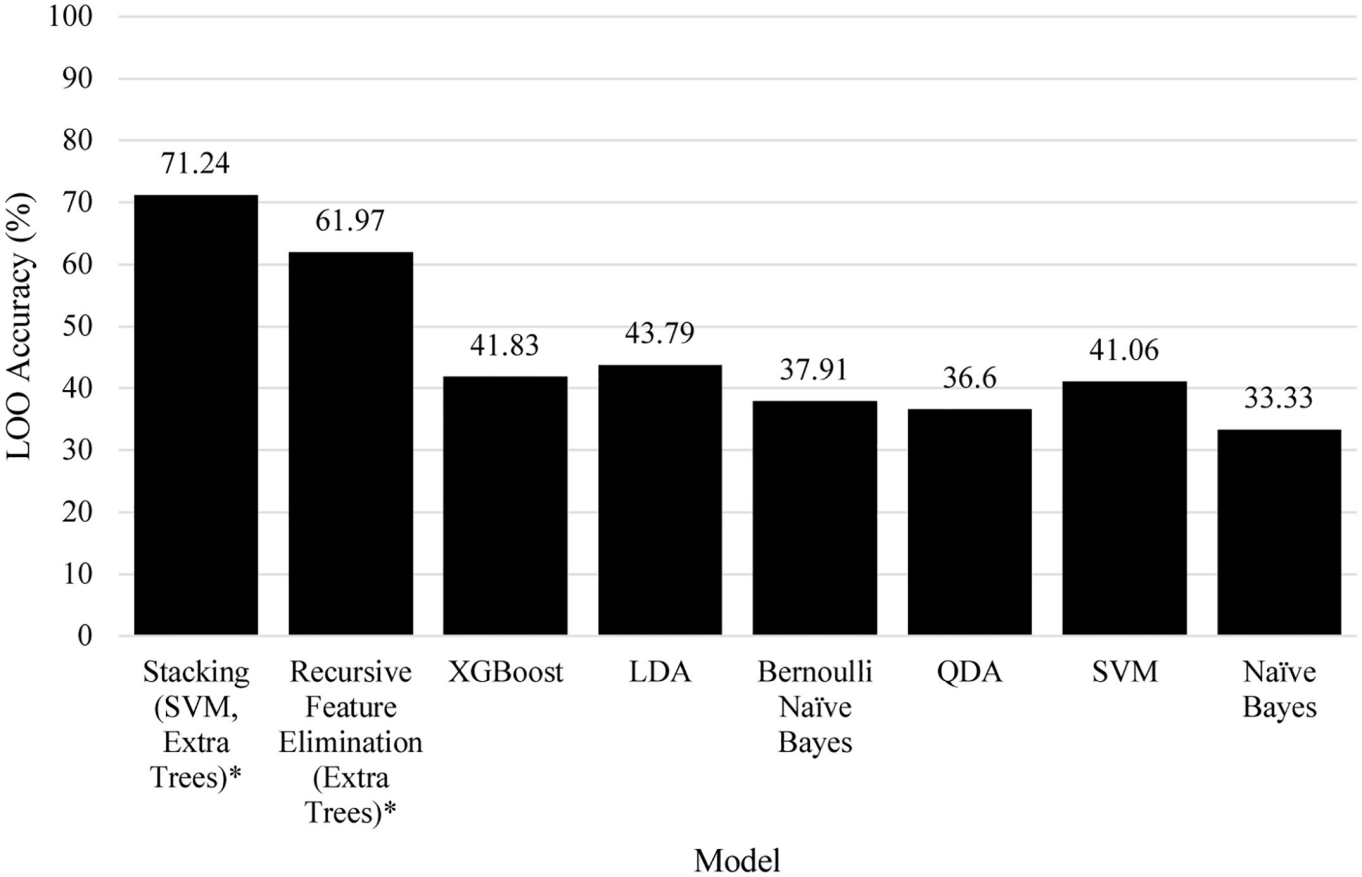
Comparison of models towards the risk of mortality, an asterisk denotes algorithms found by the genetic search algorithm.

Fig 9 shows the model comparison for risk of mortality. The difficulty of the problem can be seen with the low results achieved, with the exception of two models discovered by the genetic model search algorithm. The second best model, which utilised Extra Trees via Recursive Feature Elimination scored 61.97% and the best model found was a process of Stacking SVM and Extra Trees which had a classification ability of 71.24%.

### 3.3 Risk of inability to test

**Fig 10:**
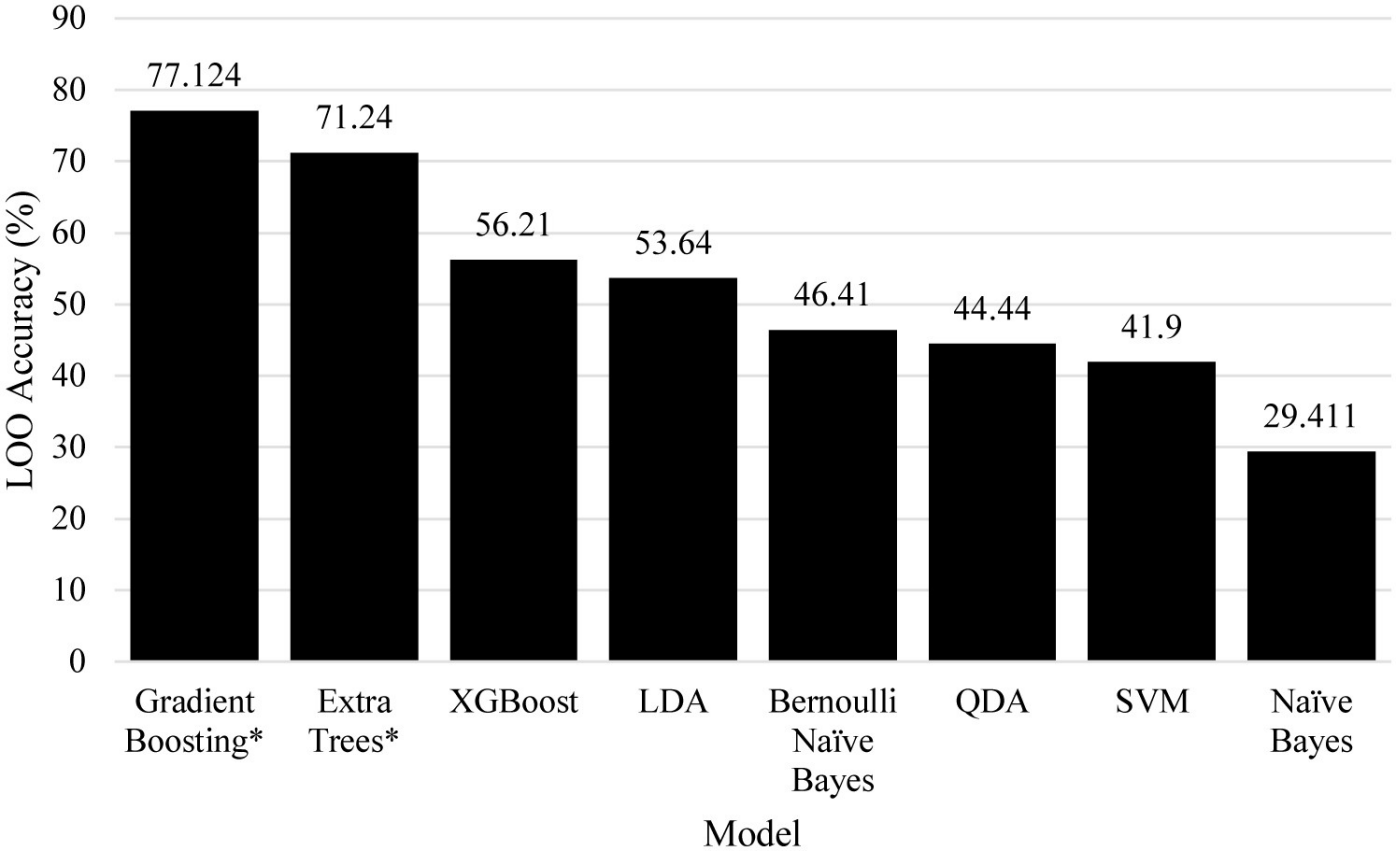
Comparison of models towards the risk of the inability to test, an asterisk denotes algorithms found by the genetic search algorithm.

Fig 6 shows the linear search of RDF estimators for the risk of inability to test. The best models were forests of 30 and 790 decision trees which both scored a LOO accuracy of 64.71%. Fig 7 shows a linear search of KNN estimators, the best was *K* = 14 which scored only 39.87% LOO accuracy. Finally, Fig 10 shows a comparison of all models benchmarked for risk of inability to test, where Gradient Boosting and Extra Trees (found by the genetic search algorithm) scored 77.12% and 71.24% LOO accuracy on the dataset.

Fig 10 shows a comparison of other models that were explored. Many solutions were quite weak, but achieving higher results in comparison to the other two problems, suggesting that the problem is a slightly less difficult one. The best algorithms, as was the case for the other problems, were also discovered by the genetic search algorithm. Unlike the previous two problems, the best models found were singular rather than either an ensemble or feature elimination pipeline, where Extra Trees scored 71.21% and Gradient Boosting scored 77.12%.

### 3.4 Comparison and interpretation

**Fig 11:**
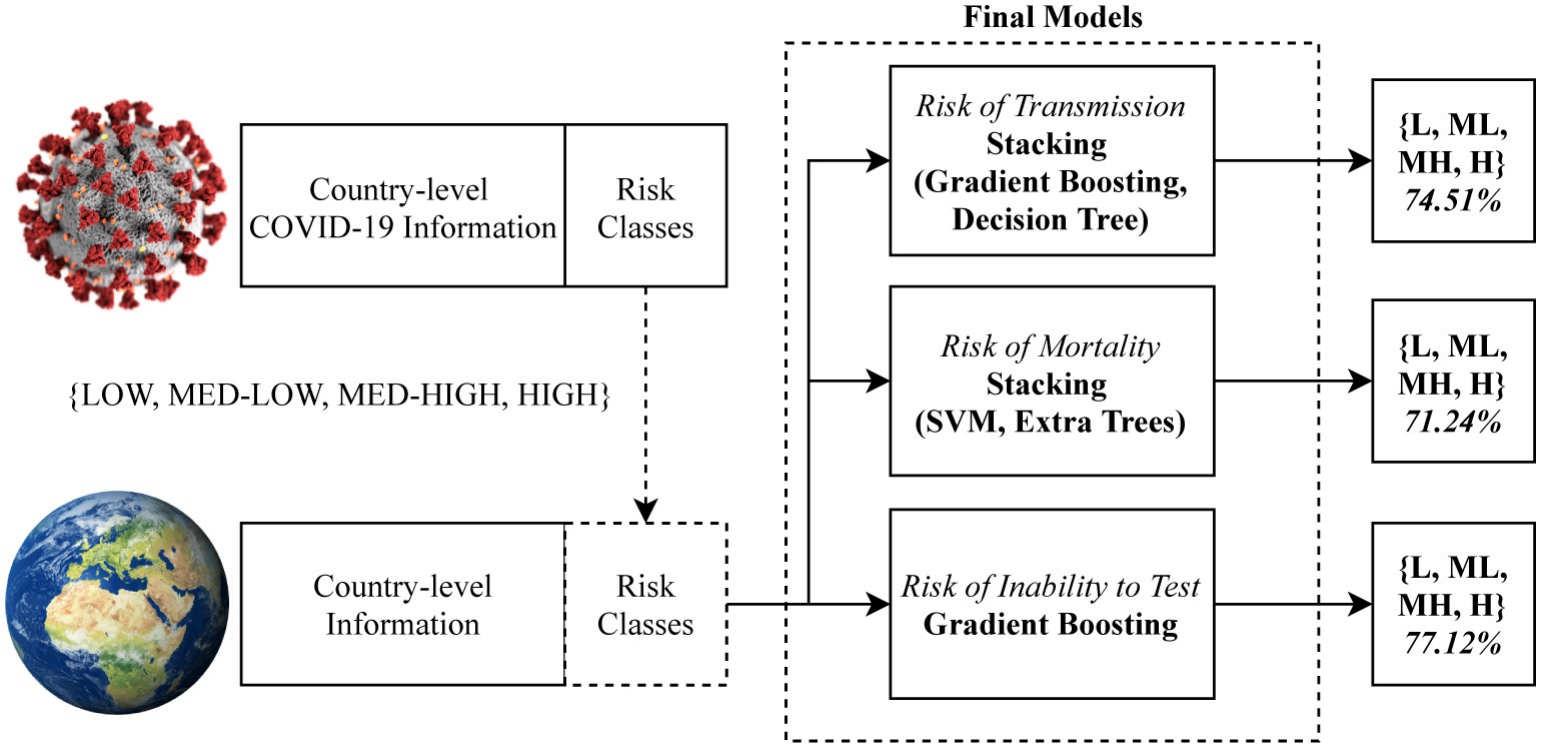
The final configuration of the framework following benchmarking experiments for each of the three risks. The best models found for each of the tasks are used in unison to predict the three risk metrics.

Following the original outline of the experiment in Figs 1 and 11 builds upon this by including the best findings from the three benchmarking experiments. The best model for Risk of Transmission was a Stacking algorithm combining Gradient Boosting and a Decision Tree for 74.51% accuracy, the best model for Risk of Mortality was a Stacking algorithm combining Support Vector Machine and Extra Trees for 71.24% accuracy, and the best model for Risk of Inability to Test was a Gradient Boosting algorithm for 77.12% accuracy. All of the best models were found by the genetic model search algorithm.

As previously discussed, the classification of risks must be interpreted relative to one another. For example, if the maps in Figs 2, 3 and 4 are observed, note that countries that do not test much also seemingly, on the surface, report fewer cases and deaths per million. On one hand, this could simply be due to the fact that there are fewer cases and thus fewer tests are required, but on the other hand, could imply that fewer tests performed have themselves led to unreported figures of the other two [49–52].

With this in mind, it is important to consider the output for *Risk of Inability to Test* in order to interpret the other two risks. In the case where the Risk of Inability to Test is towards the lower end of the spectrum, then risks for transmission and mortality are more likely to be an accurate representation of the situation. Vice versa, though, where there is a high risk of inability to test, this in itself should be considered the most descriptive risk factor for the country since there is less prior knowledge to base risks of transmission and mortality upon.

### 3.5 Application of the best models to outlier countries

Table 1 shows the predicted class values for the best models applied to each of the respective risk classification problems. Please note the discussion of interpretation in Section 3.4, where high inability to test is often coupled with lower risks of the prior two, as can be seen in Fig 5, for as of yet unknown reasons *i.e*. they could either be actually true to the pattern observed, or on the other hand, very low testing leads to naturally fewer reported cases and deaths than the actual values. Many countries bare similarity to others and so have been generalised, further outliers still such as China may not have accurately predicted labels since the population is much larger than those observed in the training data, likewise this may be the case with other geopolitical information within the outlier set.

**Table 1:** Predicted class labels for the outlier countries removed from the dataset. Observations show that high inability to test should be considered primarily, since it is often coupled with supposed ‘low risk’ of the other two classes w.r.t tests and deaths reported per million population.

Outlier Country	Risk Predictions		
	*Transmission*	*Mortality*	*Inability to Test*
***Anguilla***	2	2	2
***Burkina Faso***	1	1	4
***Cameroon***	1	1	4
***Chad***	1	1	4
China*	2	2	2
***Comoros***	3	1	4
***Repub. Congo***	1	1	4
***DRC***	1	1	4
***Eritrea***	3	1	4
***French Guiana***	1	2	3
***Guadeloupe***	3	2	2
***Guinea***	1	1	4
***Liberia***	3	1	4
***Macao***	3	2	2
***Martinique***	1	4	2
***Monaco***	4	4	1
***Nicaragua***	1	2	4
***Saint Martin***	1	3	4
***Saint Pierre Miquelon***	3	4	1
***Seychelles***	1	3	4
***Sierra Leone***	1	1	4
***Somalia***	1	1	4
***Saint Barthélemy***	1	1	2
***Sudan***	1	1	4
***Syria***	3	2	3
***Tajikistan***	1	1	4
***Tanzania***	1	1	4
***Western Sahara***	4	3	3

* Note that China has a much higher population than observed within the training data. (1—LOW, 4—HIGH).

### 3.6 Usefulness of country-level features for forecasting

In this section, we perform a preliminary exploration of how useful country-level attributes are in addition to lag-window features (seven days prior, with mean and standard deviation for days 1 − *n* via a growing lag-window). The process is implemented via a 10-fold temporal validation process (predicting future fold *k* from growing training data 1 to *k* − 1). This approach is explored for the forecasting of cases and deaths.

#### 3.6.1 Transmission (Cases)

The table within Appendix A in S1 Appendix shows the Pearson correlation coefficient of each attribute in relation to the total cases for each day. As can be expected, the most correlative feature are the cases recorded for the previous day. Interestingly, mean values of the previous two and three days have more correlation to the total cases on the current day compared to the previous day lag value alone. Gross Domestic Product and Urban Population have a weak but useful correlation for regression of the total cases. As can be expected, the singular Pearson’s correlation coefficient of each of the isolated attributes tend to be low with exception to the lag windows due to the nature of increasing growth in infections.

The tables within appendices B, C, and D in S1 Appendix detail the scores given to the attributes by Linear Regression, M5P and SVR respectively. It is observed that the rankings achieved by the lag window attributes are the same for each algorithm, and the order otherwise is relatively similar. All algorithms then rank the country at the same place above other features, which actually had a negligible correlation of 0.03. Another interesting observation is that the M5P algorithm ranks medical doctors per 1,000 population as relatively high in the ranking, second only to country when lag windows are not considered. Urban population totals are considered important by all of the algorithms, likely since this is an indication of both spread as well as a rule of thumb for total number of infected.

Table 2 shows the results for total case prediction by all of the chosen algorithms. The best algorithm achieved a RMSE of 325.66 when considering 41 features chosen by Linear Regression ranking, which were the 19 time-window attributes and 22 geopolitical or demographic attributes. This provides a decrease in RMSE of 17.08 when this algorithm only considers lags of the series, and many instances can be observed in which this metric was reduced by considering additional attributes explored within this study. The best results achieved by all of the seven algorithms considered at least two of the additional attributes, it is worth noting that the best of the best models is also the model which chose the most of the additional attributes (as well as the best SVR, which also chose 41 attributes in total).

**Table 2:** Predicting total cases: RMSE for each model via 10-fold timeseries split validation in regards to number of input features.

Inputs	Single Regressor			Voting Regressor			
	*LR*	*M5*	*SVR*	*LR, M5P*	*LR, SVR*	*M5P, SVR*	*LR, M5P, SVR*
***19***	370.22	382.94	439.52	719.09	342.74	421.82	560.88
***20***	385.85	401.72	428.66	712.86	344.47	354.51	419.36
***21***	428.45	369.37	428.66	706.07	362.26	358.07	345.73
***22***	393.85	362.10	428.66	1770.95	347.08	1117.17	682.11
***23***	352.50	1424.73	428.66	349.94	330.62	765.78	332.76
***24***	354.78	409.42	419.53	998.97	328.38	505.94	330.48
***25***	405.91	1034.08	419.53	562.31	347.90	420.13	1548.25
***26***	405.70	667.00	414.42	381.82	346.82	402.45	391.52
***27***	373.01	4171.85	414.42	1010.58	335.26	466.74	352.93
***28***	372.23	385.73	408.36	382.89	334.90	382.62	344.33
***29***	375.56	392.29	408.36	882.83	335.55	720.94	443.78
***30***	361.56	672.70	408.36	923.23	328.38	413.81	378.54
***31***	358.64	589.31	408.36	740.90	327.17	1622.69	358.53
***32***	388.40	1107.19	407.44	548.76	342.30	658.87	387.71
***33***	388.20	870.35	407.44	491.99	342.41	425.96	745.70
***34***	424.00	603.28	407.44	550.33	359.73	761.37	412.64
***35***	430.93	4742.20	407.43	521.89	362.10	873.98	639.61
***36***	446.80	486.57	407.43	2218.82	369.12	561.46	720.25
***37***	427.19	900.55	407.43	1132.58	358.86	1020.05	755.47
***38***	409.68	819.97	407.42	721.11	353.83	474.81	1028.22
***39***	562.55	4844.91	407.42	655.19	425.90	1319.26	477.66
***40***	499.31	3003.03	411.51	748.44	393.63	769.39	489.73
***41***	355.58	909.69	400.67	789.99	325.66	672.00	621.68
***42***	375.79	2177.87	400.68	2187.51	333.76	456.30	381.07
***43***	375.61	2287.24	400.68	786.87	333.28	379.21	370.89
***44***	375.61	7807.16	400.68	428.02	333.28	1237.57	445.27
***45***	375.61	862.69	400.68	2890.89	333.28	3215.68	910.77
***46***	375.61	3307.37	400.68	714.07	333.28	444.19	367.25
***Best***	352.50	362.10	400.67	349.94	325.66	354.51	330.48
***Inputs***	23	22	41	23	41	21	24

Features >19 denotes the input of geopolitical and demographic attributes selected by the model.

#### 3.6.2 Mortality (Deaths)

The table under Appendix E in S1 Appendix shows the correlation of each singular attribute towards the prediction of deaths. As can be observed, the rankings of the lag windows are the same as those for total confirmed infections described in the previous section. Otherwise, rankings are similar and differ only slightly, as well as their observed correlation.

Appendices F, G, and H in S1 Appendix detail the scores given to each attribute by the Linear Regression, M5P and Support Vector Regression algorithms respectively. As can be expected, the rankings match those of the highest correlation coefficient. Interestingly, a small change is noted within the attributes for SVR whereas quite a disparity can be seen when observing the scores given by the other two algorithms.

Table 3 shows the 189 models trained for forecasting total deaths. Similarly to the total case predictions, the best model found was within a voting ensemble of Linear Regression and SVR. Unlike total case predictions, introducing geopolitical and demographic attributes had negative effect on the result, with the best model taking only the temporal lag window features as input. Once 40 attributes were introduced, the linear regression model had an absurdly high RMSE of 2.93E+05, which since average values were taking during voting regression, also affected the ensembles that included it.

**Table 3:** Predicting total deaths: RMSE for each model via 10-fold timeseries split validation in regards to number of input features.

Inputs	Single Regressor			Voting Regressor			
	*LR*	*M5*	*SVR*	*LR, M5P*	*LR, SVR*	*M5P, SVR*	*LR, M5P, SVR*
***19***	53.73	260.87	39.64	135.28	38.98	43.95	58.65
***20***	54.15	127.71	39.64	90.35	39.20	69.42	72.83
***21***	54.54	324.05	39.55	47.52	39.32	72.09	53.40
***22***	62.54	164.76	39.75	61.18	41.79	76.75	41.99
***23***	62.80	49.82	39.30	102.89	41.98	80.73	42.61
***24***	63.20	57.32	39.30	129.20	42.16	39.82	59.99
***25***	63.51	64.68	39.30	51.91	42.32	40.07	42.14
***26***	63.70	59.86	39.29	83.76	42.39	44.16	43.57
***27***	67.78	157.60	39.29	53.84	44.45	103.40	81.46
***28***	64.97	56.71	39.29	56.87	43.04	144.12	44.45
***29***	65.01	154.23	39.29	71.13	43.07	47.01	70.55
***30***	68.31	155.96	39.29	108.55	44.56	40.63	44.66
***31***	71.17	63.24	39.30	149.10	46.03	93.16	67.95
***32***	70.85	165.38	39.30	123.68	45.82	55.12	93.80
***33***	182.45	165.99	39.35	115.57	101.66	91.77	151.65
***34***	156.53	191.46	39.35	141.29	88.61	53.06	77.72
***35***	78.32	245.34	39.36	139.82	49.58	160.23	152.77
***36***	78.29	87.45	39.36	77.59	49.65	213.65	75.67
***37***	77.20	316.22	39.39	125.16	48.90	55.41	48.91
***38***	77.20	267.06	39.39	133.38	48.90	143.74	56.71
***39***	76.02	123.55	39.62	59.53	48.55	77.34	51.08
***40***	2.93E+05	238.19	51.98	1.46E+05	1.46E+05	59.48	9.76E+04
***41***	2.93E+05	61.81	51.98	1.46E+05	1.46E+05	106.12	9.76E+04
***42***	2.93E+05	111.44	51.98	1.46E+05	1.46E+05	99.72	9.76E+04
***43***	2.93E+05	65.04	51.98	1.46E+05	1.46E+05	70.60	9.76E+04
***44***	2.93E+05	283.47	51.98	1.46E+05	1.46E+05	184.21	9.75E+04
***45***	2.93E+05	126.11	51.98	1.46E+05	1.46E+05	153.30	9.75E+04
***46***	2.93E+05	292.20	51.98	1.46E+05	1.46E+05	55.08	9.76E+04
***Best***	53.73	49.82	39.29	47.52	38.98	39.82	41.99
***Inputs***	19	23	26-30	21	19	24	22

Features >19 denotes the input of geopolitical and demographic attributes selected by the model.

### 3.7 Application of the approach to recent data

Given the nature of research and peer review, the approach in this work was formalised on the 12th of May 2020 and as such the data is over three months out of date at the time of writing (16th of September, 2020). Given this, the experiments devised in this work are re-applied to the new data. It was noted that all manual models failed to generalise with the new data. That is, a range of scores between 24.95% to 28.63% for all models, for all three risk classification problems. This is most likely due to international collaboration towards the three risk factors, and as such, country-level attributes lose classification prediction ability towards the risk factors.

With the previous successful experiments in mind, this argues that risk classification would be more useful when performed prior to the situation unfolding, given that country-level information is seemingly more important at this stage when compared to the current post-peak climate. Though much weaker results are now observed, this could in fact be viewed as a positive situation, given that country-level data i.e. *who you are and where you are from* no longer impacts risk as it was observed to in the initial experiments performed in May 2020. It has been noted during mid-2020 that organisations such as the United Nations and World Health Organisation have implemented and released humanitarian packages to Lower Economically Developed Countries (LEDCs) [53–55]. It has also been noted that many healthcare professionals returned to their native countries (often also LEDCs) in order to aid in tackling the virus [56]. The positive effects of these factors likely contribute towards the reason why country-level information was useful for risk classification earlier in the pandemic, but are less-so later on post-peak.

## 4 Future work and conclusion

With the nature of the data streaming from the ongoing pandemic with regards to the time taken to run model benchmarks, the largest and most obvious limitation to this study is that the models are constantly going out of date by the day, since more up to date data is constantly becoming available. It is for this reason that the models should be updated at a later date, and the statistical differences that occur, if any, noted. Secondly, though relatively good results were found through a complex process of genetic optimisation, further models could be explored in order to possibly reach better results than the final models in this study. Finally, the interpretation that is required as aforementioned, i.e. that the risk of inability to test is the most important metric and possibly enables the other two for interpretation, suggests that the ternary approach followed could be better optimised through a unified approach. That is, one singular *“metric of risk”* that is calculated via the three metrics explored in this work as separate problems. Prior to this study, a metric of (*c* + *d*)/*t* was explored (where *c*, *d*, and *t* denote cases, deaths, and tests respectively, all with regards to per million population), but this metric is, at this point, impossible to classify.

The *K*% method was used to divide the continuous features into four bins where *K* = 25. Other methods of binning such as MDL [57], CAIM, CACC, and Ameva [58] could also be explored and benchmarked in future experiments.

To conclude, the main hypothesis that this work has argued in favour of is that geopolitical and demographic attributes at the country-level hold value in terms of classifying risk produced by the COVID-19 dataset. This was shown when the four class distribution which was close to equal (’HIGH’ was 1.2% larger than the other classes) could be classified far above the approximate 25% class distribution through LOO CV. Though this is observably possible from the results presented in this study, it is worth noting that the classification problem proved extremely difficult for many powerful machine learning techniques, which often scored around only 40%, and a genetic search had to be followed in order to devise complex strategies of ensemble and hyperparameter optimisation in order to achieve better results at 74.51%, 71.24%, and 77.12% for the three problems. Future work aims to keep the data up to date to the point at which the pandemic is over, and also to explore other methods of solving the issue of risk and preparedness classification through a more unified approach as well as through stronger machine learning models, if possible.

## Supporting information

S1 Appendix (PDF)

S1 Fig (TIF)

S2 Fig (TIF)

S3 Fig (TIF)
