# Supplementary material for: Country-level pandemic risk and preparedness classification based on COVID-19 data: A machine learning approach
Source: PLoS One. 2020 Oct 28;15(10):e0241332. doi: 10.1371/journal.pone.0241332 (PMC7592809; doi:10.1371/journal.pone.0241332)
Supplement: S1 Appendix — (PDF) [file pone.0241332.s001.pdf]

A Pearson's Correlation Coefficient for each isolated attribute towards Total Cases

**Attribute**	**Correlation**
LAG_TOTAL-CASES-1	0.99948
LAG_TOTAL-CASES-MEAN-1-2	0.99886
LAG_TOTAL-CASES-MEAN-1-3	0.998
LAG_TOTAL-CASES-2	0.99792
LAG_TOTAL-CASES-MEAN-1-4	0.9969
LAG_TOTAL-CASES-MEAN-1-5	0.99556
LAG_TOTAL-CASES-3	0.99523
LAG_TOTAL-CASES-MEAN-1-6	0.99398
LAG_TOTAL-CASES-MEAN-1-7	0.99218
LAG_TOTAL-CASES-4	0.99131
LAG_TOTAL-CASES-5	0.98602
LAG_TOTAL-CASES-6	0.97928
LAG_TOTAL-CASES-7	0.97096
LAG_TOTAL-CASES-STDDEV-1-7	0.91364
LAG_TOTAL-CASES-STDDEV-1-6	0.90624
LAG_TOTAL-CASES-STDDEV-1-5	0.89902
LAG_TOTAL-CASES-STDDEV-1-4	0.89172
LAG_TOTAL-CASES-STDDEV-1-3	0.8838
LAG_TOTAL-CASES-STDDEV-1-2	0.87316
GDP-USD-2019-IMF	0.42584
URBAN-POP	0.24527
AREA-KM2	0.19366
POPULATION	0.1819
MONTH	0.17737
SERVICE-%-GDP	0.10593
MEDIAN-AGE	0.1003
NURSING-AND-MIDWIFERY-PERSONNEL-(PER-10000)	0.07837
OBESITY-PREVALENCE-2016	0.07483
LITERACY-%	0.07457
UN-REGIONAL-GROUP	0.05916
ARABLE-LAND-%	0.05392
MEDICAL-DOCTORS-(PER-10000)	0.04007
COUNTRY	0.03861
CLIMATE-CATEGORY	0.03833
NET-MIGRATION	0.0318
DEATHRATE-(PER-1000)	0.02961
URBAN-POP%	0.02936
CROP-LAND-%	0.00697
TOBACCO-PREVALENCE-2015	-0.00928
DAY	-0.03282
POP-DENSITY-KM2	-0.03776
OTHER-LAND-%	-0.04997
INFANT-MORTALITY-(PER-1000-BIRTHS)	-0.06852
AGRICULTURE-%-GDP	-0.07354
INDUSTRY-%-GDP	-0.07616
BIRTHRATE-(PER-1000)	-0.08499

B Worthiness scores of each isolated attribute towards Total Cases according to Linear Regression

Attribute	Score
LAG_TOTAL-CASES-1	37206.701
LAG_TOTAL-CASES-MEAN-1-2	36607.5155
LAG_TOTAL-CASES-MEAN-1-3	36006.1913
LAG_TOTAL-CASES-2	35958.8577
LAG_TOTAL-CASES-MEAN-1-4	35403.6913
LAG_TOTAL-CASES-MEAN-1-5	34802.5844
LAG_TOTAL-CASES-3	34678.0896
LAG_TOTAL-CASES-MEAN-1-6	34204.4299
LAG_TOTAL-CASES-MEAN-1-7	33611.968
LAG_TOTAL-CASES-4	33353.2911
LAG_TOTAL-CASES-5	31992.3457
LAG_TOTAL-CASES-6	30594.2777
LAG_TOTAL-CASES-7	29175.4949
LAG_TOTAL-CASES-STDDEV-1-7	22522.4518
LAG_TOTAL-CASES-STDDEV-1-6	21870.8786
LAG_TOTAL-CASES-STDDEV-1-5	21269.9635
LAG_TOTAL-CASES-STDDEV-1-4	20698.2028
LAG_TOTAL-CASES-STDDEV-1-3	20106.7742
LAG_TOTAL-CASES-STDDEV-1-2	19309.0564
COUNTRY	4665.2192
GDP-USD-2019-IMF	3531.7111
UN-REGIONAL-GROUP	1601.0997
URBAN-POP	1151.4407
AREA-KM2	686.99
POPULATION	626.901
MONTH	597.8549
SERVICE-%-GDP	203.237
MEDIAN-AGE	189.8983
BIRTHRATE-(PER-1000)	135.914
NURSING-AND-MIDWIFERY-PERSONNEL-(PER-10000)	109.268
LITERACY-%	104.3904
INDUSTRY-%-GDP	103.4102
AGRICULTURE-%-GDP	98.7466
OBESITY-PREVALENCE-2016	89.216
INFANT-MORTALITY-(PER-1000-BIRTHS)	86.0903
ARABLE-LAND-%	54.1728
OTHER-LAND-%	44.6811
POP-DENSITY-KM2	26.7876
MEDICAL-DOCTORS-(PER-10000)	25.5937
CLIMATE-CATEGORY	19.4814
NET-MIGRATION	15.7625
DEATHRATE-(PER-1000)	14.5646
URBAN-POP%	13.8685
DAY	9.8493
TOBACCO-PREVALENCE-2015	0
CROP-LAND-%	0

C Worthiness scores of each isolated attribute towards Total Cases according to the M5P algorithm

Attribute	Score
LAG_TOTAL-CASES-1	37134.73753
LAG_TOTAL-CASES-MEAN-1-2	36663.93786
LAG_TOTAL-CASES-MEAN-1-3	36247.28586
LAG_TOTAL-CASES-2	36203.2862
LAG_TOTAL-CASES-MEAN-1-4	35586.09743
LAG_TOTAL-CASES-MEAN-1-5	35190.87717
LAG_TOTAL-CASES-3	35122.90702
LAG_TOTAL-CASES-MEAN-1-6	34707.62698
LAG_TOTAL-CASES-MEAN-1-7	34132.99247
LAG_TOTAL-CASES-4	33936.54895
LAG_TOTAL-CASES-5	32651.27439
LAG_TOTAL-CASES-6	31400.59122
LAG_TOTAL-CASES-7	30309.1689
LAG_TOTAL-CASES-STDDEV-1-7	22646.83088
LAG_TOTAL-CASES-STDDEV-1-6	22104.08242
LAG_TOTAL-CASES-STDDEV-1-5	21565.30804
LAG_TOTAL-CASES-STDDEV-1-4	20959.74051
LAG_TOTAL-CASES-STDDEV-1-3	20214.21775
LAG_TOTAL-CASES-STDDEV-1-2	19551.86468
COUNTRY	4660.37328
MEDICAL-DOCTORS-(PER-10000)	4583.2974
BIRTHRATE-(PER-1000)	4555.15289
URBAN-POP	4553.92636
ARABLE-LAND-%	4540.24245
DEATHRATE-(PER-1000)	4529.48358
NET-MIGRATION	4512.49074
POPULATION	4491.58778
CROP-LAND-%	4486.14752
AREA-KM2	4438.68472
MEDIAN-AGE	4384.98746
TOBACCO-PREVALENCE-2015	4339.34344
NURSING-AND-MIDWIFERY-PERSONNEL-(PER-10000)	4335.73292
URBAN-POP%	4314.18357
INFANT-MORTALITY-(PER-1000-BIRTHS)	4289.45891
POP-DENSITY-KM2	4107.75006
OTHER-LAND-%	3968.00674
SERVICE-%-GDP	3744.12062
GDP-USD-2019-IMF	3531.71113
OBESITY-PREVALENCE-2016	3105.60779
INDUSTRY-%-GDP	2920.73294
LITERACY-%	1422.0776
AGRICULTURE-%-GDP	1251.56814
MONTH	825.50979
UN-REGIONAL-GROUP	777.71847
CLIMATE-CATEGORY	398.45307
DAY	136.29896

D Worthiness scores of each attribute towards Total Cases according to Support Vector Regression

Attribute	Score
LAG_TOTAL-CASES-1	35615.4005
LAG_TOTAL-CASES-MEAN-1-2	35555.603
LAG_TOTAL-CASES-MEAN-1-3	35493.0074
LAG_TOTAL-CASES-2	354881.7026
LAG_TOTAL-CASES-MEAN-1-4	35432.60164
LAG_TOTAL-CASES-MEAN-1-5	35371.79469
LAG_TOTAL-CASES-3	35357.94243
LAG_TOTAL-CASES-MEAN-1-6	35310.31758
LAG_TOTAL-CASES-MEAN-1-7	35249.36718
LAG_TOTAL-CASES-4	35221.41054
LAG_TOTAL-CASES-5	35079.82154
LAG_TOTAL-CASES-6	34932.77621
LAG_TOTAL-CASES-7	34780.39
LAG_TOTAL-CASES-STDDEV-1-7	34090.12674
LAG_TOTAL-CASES-STDDEV-1-6	34030.71485
LAG_TOTAL-CASES-STDDEV-1-5	33973.13882
LAG_TOTAL-CASES-STDDEV-1-4	33921.5182
LAG_TOTAL-CASES-STDDEV-1-3	33869.53804
LAG_TOTAL-CASES-STDDEV-1-2	33781.76575
COUNTRY	31914.01019
URBAN-POP	31857.31578
GDP-USD-2019-IMF	31849.98511
MONTH	31839.21394
POPULATION	31831.33164
CROP-LAND-%	31826.45375
DAY	31826.3678
LITERACY-%	31826.36209
URBAN-POP%	31826.33466
INFANT-MORTALITY-(PER-1000-BIRTHS)	31826.28948
MEDIAN-AGE	31826.27703
OBESITY-PREVALENCE-2016	31826.26912
ARABLE-LAND-%	31826.26737
OTHER-LAND-%	31826.23384
POP-DENSITY-KM2	31826.21591
MEDICAL-DOCTORS-(PER-10000)	31826.18814
BIRTHRATE-(PER-1000)	31826.04445
SERVICE-%-GDP	31826.00056
INDUSTRY-%-GDP	31825.93348
NURSING-AND-MIDWIFERY-PERSONNEL-(PER-10000)	31825.92749
CLIMATE-CATEGORY	31825.85446
TOBACCO-PREVALENCE-2015	31825.82837
AGRICULTURE-%-GDP	31825.82704
DEATHRATE-(PER-1000)	31825.29397
NET-MIGRATION	31825.24368
AREA-KM2	31824.32713
UN-REGIONAL-GROUP	31820.75339

E Pearson's Correlation Coefficient for each isolated attribute towards Total Deaths

Attribute	Correlation
LAG_TOTAL-DEATHS-1	0.999
LAG_TOTAL-DEATHS-MEAN-1-2	0.9981
LAG_TOTAL-DEATHS-MEAN-1-3	0.9968
LAG_TOTAL-DEATHS-2	0.9966
LAG_TOTAL-DEATHS-MEAN-1-4	0.9953
LAG_TOTAL-DEATHS-MEAN-1-5	0.9935
LAG_TOTAL-DEATHS-3	0.9926
LAG_TOTAL-DEATHS-MEAN-1-6	0.9914
LAG_TOTAL-DEATHS-MEAN-1-7	0.9891
LAG_TOTAL-DEATHS-4	0.987
LAG_TOTAL-DEATHS-5	0.9802
LAG_TOTAL-DEATHS-6	0.9717
LAG_TOTAL-DEATHS-7	0.9611
LAG_TOTAL-DEATHS-STDDEV-1-7	0.92
LAG_TOTAL-DEATHS-STDDEV-1-6	0.9119
LAG_TOTAL-DEATHS-STDDEV-1-5	0.9028
LAG_TOTAL-DEATHS-STDDEV-1-4	0.8918
LAG_TOTAL-DEATHS-STDDEV-1-3	0.8774
LAG_TOTAL-DEATHS-STDDEV-1-2	0.8489
GDP-USD-2019-IMF	0.2581
MONTH	0.1841
URBAN-POP	0.1349
MEDIAN-AGE	0.1331
SERVICE-%-GDP	0.1278
POPULATION	0.0939
AREA-KM2	0.0849
UN-REGIONAL-GROUP	0.0835
CROP-LAND-%	0.0821
ARABLE-LAND-%	0.079
LITERACY-%	0.0776
MEDICAL-DOCTORS-(PER-10000)	0.0717
OBESITY-PREVALENCE-2016	0.0673
DEATHRATE-(PER-1000)	0.0557
NURSING-AND-MIDWIFERY-PERSONNEL-(PER-10000)	0.0464
COUNTRY	0.039
URBAN-POP%	0.0367
NET-MIGRATION	0.0224
CLIMATE-CATEGORY	0.0139
TOBACCO-PREVALENCE-2015	-0.0207
DAY	-0.0324
POP-DENSITY-KM2	-0.0335
INFANT-MORTALITY-(PER-1000-BIRTHS)	-0.0854
AGRICULTURE-%-GDP	-0.0874
OTHER-LAND-%	-0.0927
INDUSTRY-%-GDP	-0.0933
BIRTHRATE-(PER-1000)	-0.1007

F Worthiness scores of each isolated attribute towards Total Deaths according to Linear Regression

**Attribute**	**Score**
LAG_TOTAL-DEATHS-1	2311.6316
LAG_TOTAL-DEATHS-MEAN-1-2	2270.1914
LAG_TOTAL-DEATHS-MEAN-1-3	2226.3242
LAG_TOTAL-DEATHS-2	2218.6709
LAG_TOTAL-DEATHS-MEAN-1-4	2183.3483
LAG_TOTAL-DEATHS-MEAN-1-5	2141.3606
LAG_TOTAL-DEATHS-3	2122.3639
LAG_TOTAL-DEATHS-MEAN-1-6	2100.3379
LAG_TOTAL-DEATHS-MEAN-1-7	2059.7627
LAG_TOTAL-DEATHS-4	2026.3775
LAG_TOTAL-DEATHS-5	1934.7969
LAG_TOTAL-DEATHS-6	1842.0915
LAG_TOTAL-DEATHS-7	1744.0319
LAG_TOTAL-DEATHS-STDDEV-1-7	1466.0419
LAG_TOTAL-DEATHS-STDDEV-1-6	1419.844
LAG_TOTAL-DEATHS-STDDEV-1-5	1369.5553
LAG_TOTAL-DEATHS-STDDEV-1-4	1313.8293
LAG_TOTAL-DEATHS-STDDEV-1-3	1241.3134
LAG_TOTAL-DEATHS-STDDEV-1-2	1119.5626
COUNTRY	274.6036
GDP-USD-2019-IMF	77.2337
UN-REGIONAL-GROUP	66.7914
MONTH	40.6414
URBAN-POP	21.1301
MEDIAN-AGE	20.8482
SERVICE-%-GDP	19.0241
BIRTHRATE-(PER-1000)	11.9017
POPULATION	10.088
INDUSTRY-%-GDP	10.059
OTHER-LAND-%	10.027
AGRICULTURE-%-GDP	8.8834
INFANT-MORTALITY-(PER-1000-BIRTHS)	8.4657
ARABLE-LAND-%	7.3922
AREA-KM2	7.3879
LITERACY-%	7.0868
CROP-LAND-%	6.4201
MEDICAL-DOCTORS-(PER-10000)	5.7095
OBESITY-PREVALENCE-2016	4.8325
DEATHRATE-(PER-1000)	3.5104
NURSING-AND-MIDWIFERY-PERSONNEL-(PER-10000)	2.1441
URBAN-POP%	1.4037
POP-DENSITY-KM2	1.3382
DAY	0.6087
TOBACCO-PREVALENCE-2015	-0.1345
NET-MIGRATION	-0.1459
CLIMATE-CATEGORY	-1.1914

G Worthiness scores of each isolated attribute towards Total Deaths according to the M5P algorithm

Attribute	Score
LAG_TOTAL-DEATHS-1	2311.5648
LAG_TOTAL-DEATHS-MEAN-1-2	2277.9243
LAG_TOTAL-DEATHS-MEAN-1-3	2238.0698
LAG_TOTAL-DEATHS-2	2234.6567
LAG_TOTAL-DEATHS-MEAN-1-4	2200.9028
LAG_TOTAL-DEATHS-MEAN-1-5	2168.7158
LAG_TOTAL-DEATHS-3	2143.2901
LAG_TOTAL-DEATHS-MEAN-1-6	2114.842
LAG_TOTAL-DEATHS-MEAN-1-7	2071.5819
LAG_TOTAL-DEATHS-4	2043.2872
LAG_TOTAL-DEATHS-5	1958.5161
LAG_TOTAL-DEATHS-6	1884.6156
LAG_TOTAL-DEATHS-7	1802.6213
LAG_TOTAL-DEATHS-STDDEV-1-7	1582.9358
LAG_TOTAL-DEATHS-STDDEV-1-6	1531.9202
LAG_TOTAL-DEATHS-STDDEV-1-5	1519.7093
LAG_TOTAL-DEATHS-STDDEV-1-4	1457.102
LAG_TOTAL-DEATHS-STDDEV-1-3	1370.7645
LAG_TOTAL-DEATHS-STDDEV-1-2	1354.3583
COUNTRY	274.6155
NURSING-AND-MIDWIFERY-PERSONNEL-(PER-10000)	263.9846
DEATHRATE-(PER-1000)	263.9541
POPULATION	263.3408
CROP-LAND-%	261.063
MEDICAL-DOCTORS-(PER-10000)	260.1356
AREA-KM2	259.7726
GDP-USD-2019-IMF	257.4675
NET-MIGRATION	254.6027
POP-DENSITY-KM2	254.3377
URBAN-POP	251.8644
BIRTHRATE-(PER-1000)	250.8911
MEDIAN-AGE	244.8103
OTHER-LAND-%	240.1885
TOBACCO-PREVALENCE-2015	236.1389
INFANT-MORTALITY-(PER-1000-BIRTHS)	232.9417
ARABLE-LAND-%	207.0676
URBAN-POP%	197.8494
SERVICE-%-GDP	194.2973
OBESITY-PREVALENCE-2016	131.9728
INDUSTRY-%-GDP	98.598
LITERACY-%	76.2611
UN-REGIONAL-GROUP	62.7297
MONTH	57.6035
AGRICULTURE-%-GDP	56.3932
CLIMATE-CATEGORY	15.7606
DAY	12.4268

H Worthiness scores of each attribute towards Total Deaths according to Support Vector Regression

Attribute	Score
LAG_TOTAL-DEATHS-1	18807.512
LAG_TOTAL-DEATHS-MEAN-1-2	18767.7191
LAG_TOTAL-DEATHS-MEAN-1-3	18723.1037
LAG_TOTAL-DEATHS-2	18714.5615
LAG_TOTAL-DEATHS-MEAN-1-4	18680.1828
LAG_TOTAL-DEATHS-MEAN-1-5	18637.6544
LAG_TOTAL-DEATHS-3	18619.2813
LAG_TOTAL-DEATHS-MEAN-1-6	18595.0416
LAG_TOTAL-DEATHS-MEAN-1-7	18554.6694
LAG_TOTAL-DEATHS-4	18519.1105
LAG_TOTAL-DEATHS-5	18424.2527
LAG_TOTAL-DEATHS-6	18331.3825
LAG_TOTAL-DEATHS-7	18231.8148
LAG_TOTAL-DEATHS-STDDEV-1-7	17918.0277
LAG_TOTAL-DEATHS-STDDEV-1-6	17874.6538
LAG_TOTAL-DEATHS-STDDEV-1-5	17821.7866
LAG_TOTAL-DEATHS-STDDEV-1-4	17767.3844
LAG_TOTAL-DEATHS-STDDEV-1-3	17712.9526
LAG_TOTAL-DEATHS-STDDEV-1-2	17607.9313
COUNTRY	16496.2543
URBAN-POP	16463.8353
GDP-USD-2019-IMF	16462.201
MONTH	16461.9899
POP-DENSITY-KM2	16461.8997
DAY	16461.7064
MEDICAL-DOCTORS-(PER-10000)	16461.4527
UN-REGIONAL-GROUP	16461.4101
SERVICE-%-GDP	16461.3989
MEDIAN-AGE	16461.3557
OTHER-LAND-%	16461.3514
URBAN-POP%	16461.3478
NURSING-AND-MIDWIFERY-PERSONNEL-(PER-10000)	16461.3001
INDUSTRY-%-GDP	16461.2699
LITERACY-%	16461.2593
CLIMATE-CATEGORY	16461.2529
AGRICULTURE-%-GDP	16461.2218
OBESITY-PREVALENCE-2016	16461.1998
INFANT-MORTALITY-(PER-1000-BIRTHS)	16461.1568
CROP-LAND-%	16461.1114
BIRTHRATE-(PER-1000)	16461.0429
ARABLE-LAND-%	16460.9936
TOBACCO-PREVALENCE-2015	16460.9202
DEATHRATE-(PER-1000)	16460.6551
NET-MIGRATION	16460.5256
AREA-KM2	16458.954
POPULATION	16458.3098
