# Supplementary figures and images for: Country-level pandemic risk and preparedness classification based on COVID-19 data: A machine learning approach
Source: PLoS One. 2020 Oct 28;15(10):e0241332. doi: 10.1371/journal.pone.0241332 (PMC7592809; doi:10.1371/journal.pone.0241332)

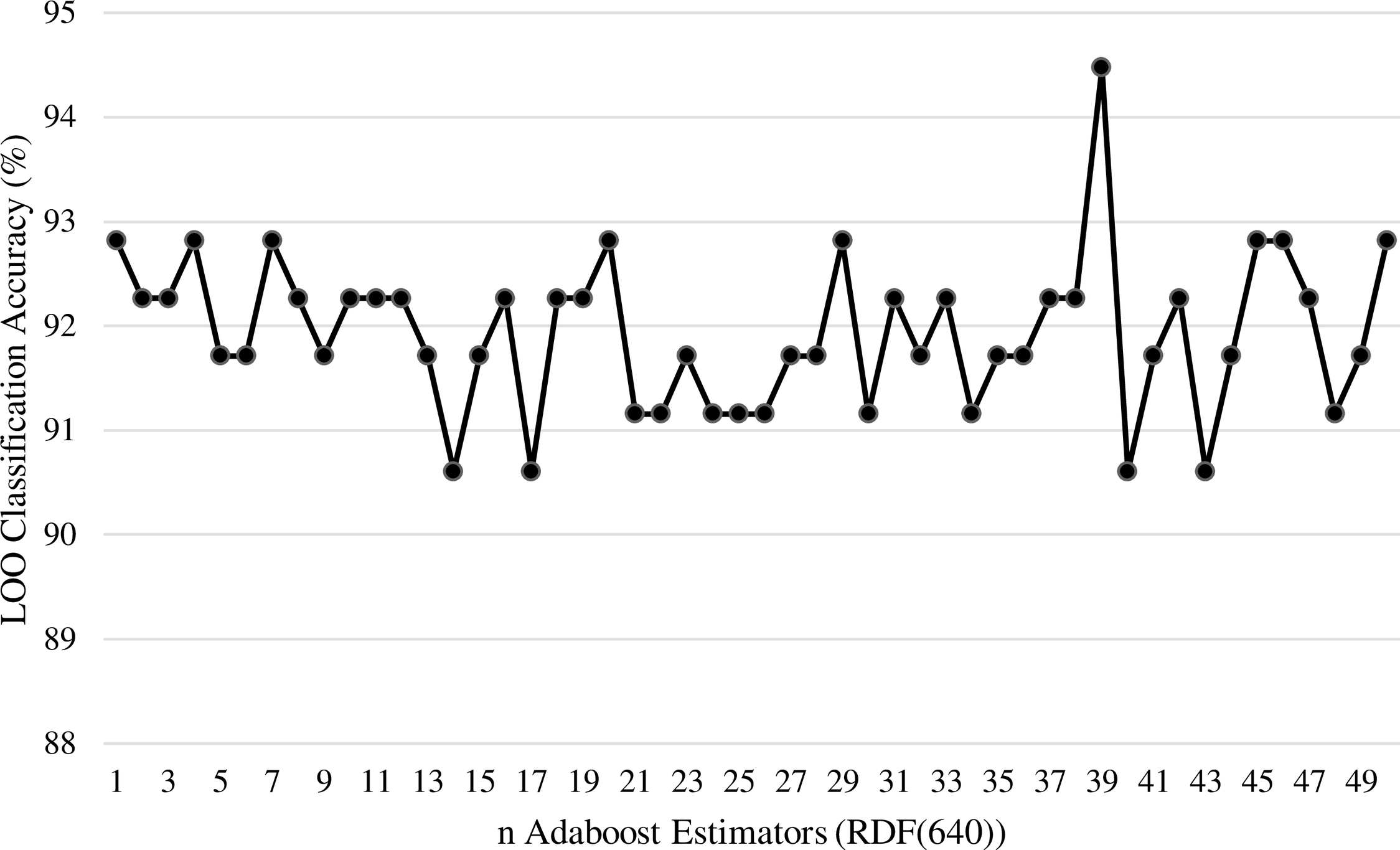

Supplement: S1 Fig — (TIF) [file pone.0241332.s002.tif]

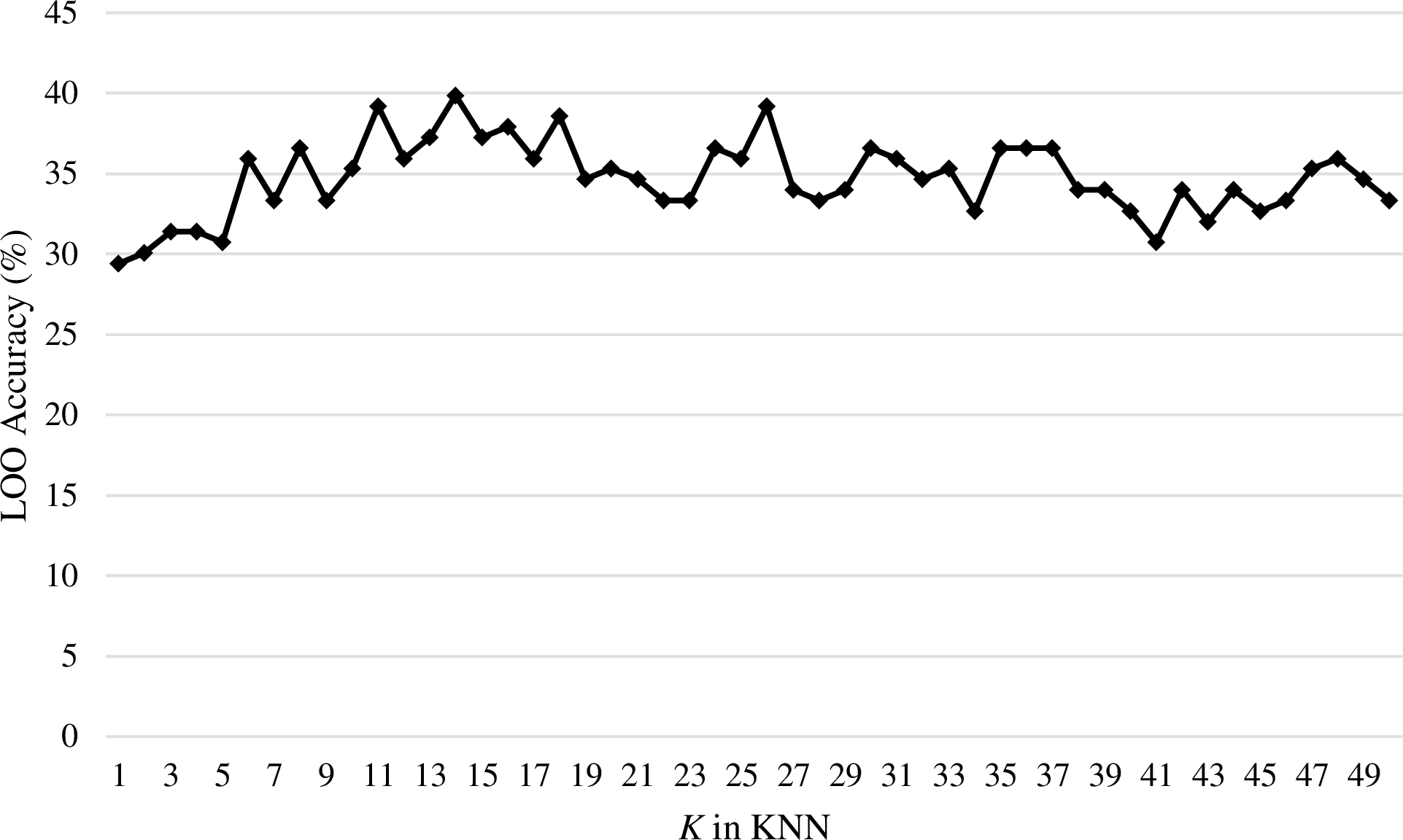

Supplement: S2 Fig — (TIF) [file pone.0241332.s003.tif]

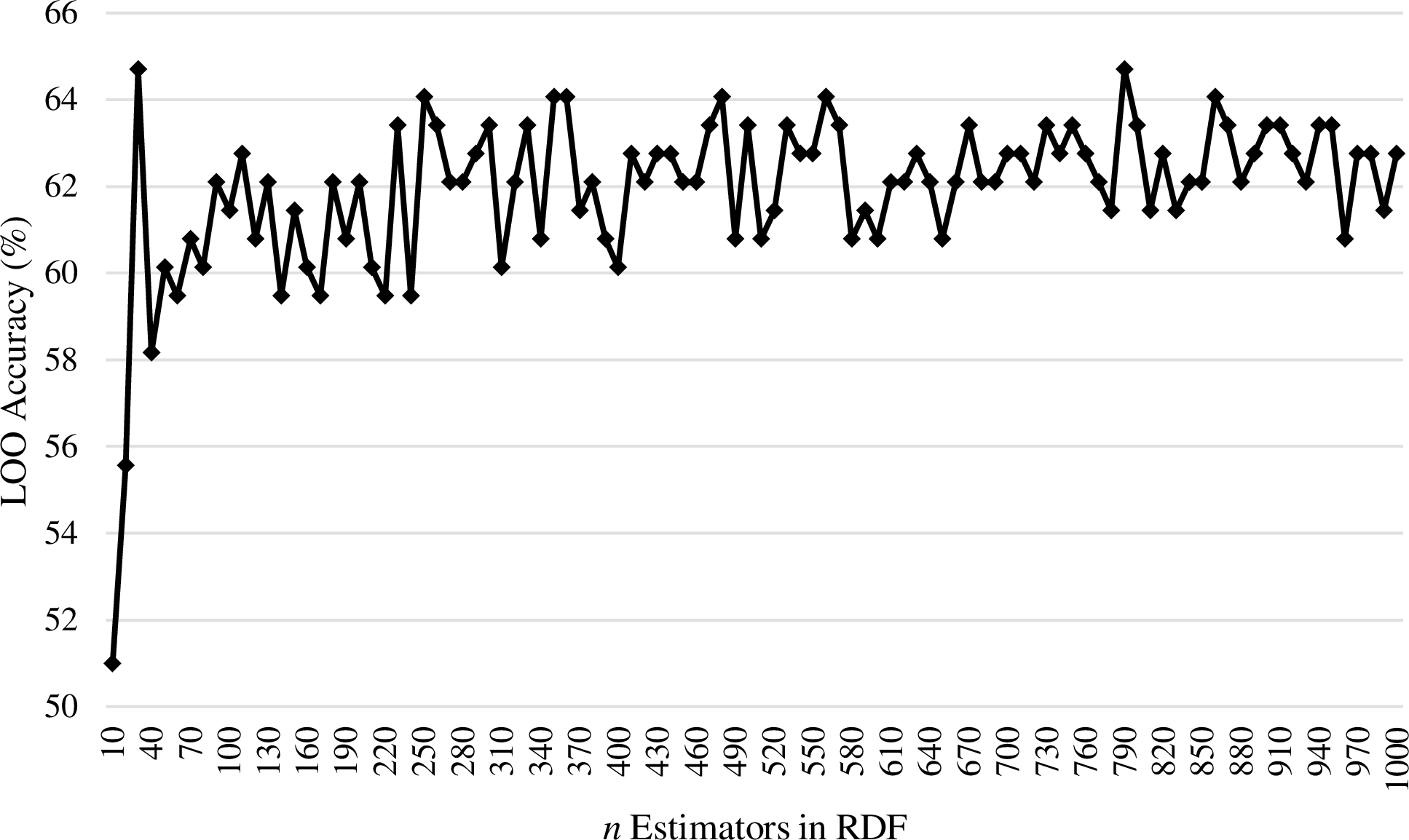

Supplement: S3 Fig — (TIF) [file pone.0241332.s004.tif]
